# Autothermal hydrogen production *via* oxidative decomposition of ammonia over vacancy-engineered Ru/CeO_2_ prepared by NaBH_4_ liquid-phase reduction

**DOI:** 10.1039/d6ra06193a

**Published:** 2026-10-05

**Authors:** Soohang Lee, Seok-Ho Lee, Minseon Park, Tae Hyun Kim, In Hye Kwak, Hyeon Ih Ryu, Na Young Kim, Jin Suk Chung, Byung Jin Lee, Kwan-Young Lee

**Affiliations:** a Department of Chemical and Biological Engineering, Korea University 145 Anam-ro Seongbuk-gu Seoul 02841 Republic of Korea kylee@korea.ac.kr; b Strategic Research Center for Clean Hydrogen Storage and Utilization, Korea Institute of Science and Technology Seoul 02792 Republic of Korea; c School of Energy and Chemical Engineering, University of Ulsan 93 Daehak-ro, Nam-gu Ulsan 44610 Republic of Korea byungjlee@ulsan.ac.kr; d Strategic Research Center for Smart Battery, Korea Basic Science Institute Daejeon 34133 Korea

## Abstract

Ammonia is a promising hydrogen carrier owing to its high volumetric hydrogen density. Oxidative decomposition of ammonia (ODA) enables autothermal hydrogen production by using the heat released from partial ammonia combustion. However, because this heat is supplied by combusting part of the feed, thermal self-sufficiency and hydrogen yield are intrinsically coupled: at NH_3_/O_2_ = 4 the oxidized fraction caps the attainable H_2_ yield at 66.7%. The central catalytic challenge is therefore to maximize decomposition activity at a fixed combustion fraction. Here we activate Ru/CeO_2_ by sodium borohydride (NaBH_4_)-mediated liquid-phase reduction instead of conventional high-temperature H_2_ reduction, thereby decoupling oxygen-vacancy formation from Ru sintering. The liquid-phase-reduced catalyst (LPR) retained a Ru particle size comparable to that of the mildly reduced catalyst (LT) while exhibiting a markedly higher vacancy concentration by XPS and Ce M_4,5_-edge EELS. N_2_-TPD shows that LPR releases nitrogen at the lowest temperature (370 °C *versus* 430 °C for LT), consistent with weakened Ru–N binding at the rate-limiting recombinative desorption step, and H_2_-TPD with CO/H_2_ pulse chemisorption indicates enhanced hydrogen spillover to the support. Thermochemical analysis shows that ODA becomes thermally neutral when about 12.7% of the ammonia feed is oxidized (O_2_/NH_3_ = 0.095). At O_2_/NH_3_ = 0.25, LPR delivered a 26.4% H_2_ yield *versus* 20.6% for LT, corresponding to 39.6% and 30.9% of the 66.7% theoretical ceiling. A concept-level techno-economic analysis gives a levelized hydrogen cost of $9.6 kg^−1^-H_2_ at the experimental yield, dominated by the ammonia feedstock.

## Abstract 2

Ammonia is a promising hydrogen carrier owing to its high volumetric hydrogen density.

## Graphical abstract

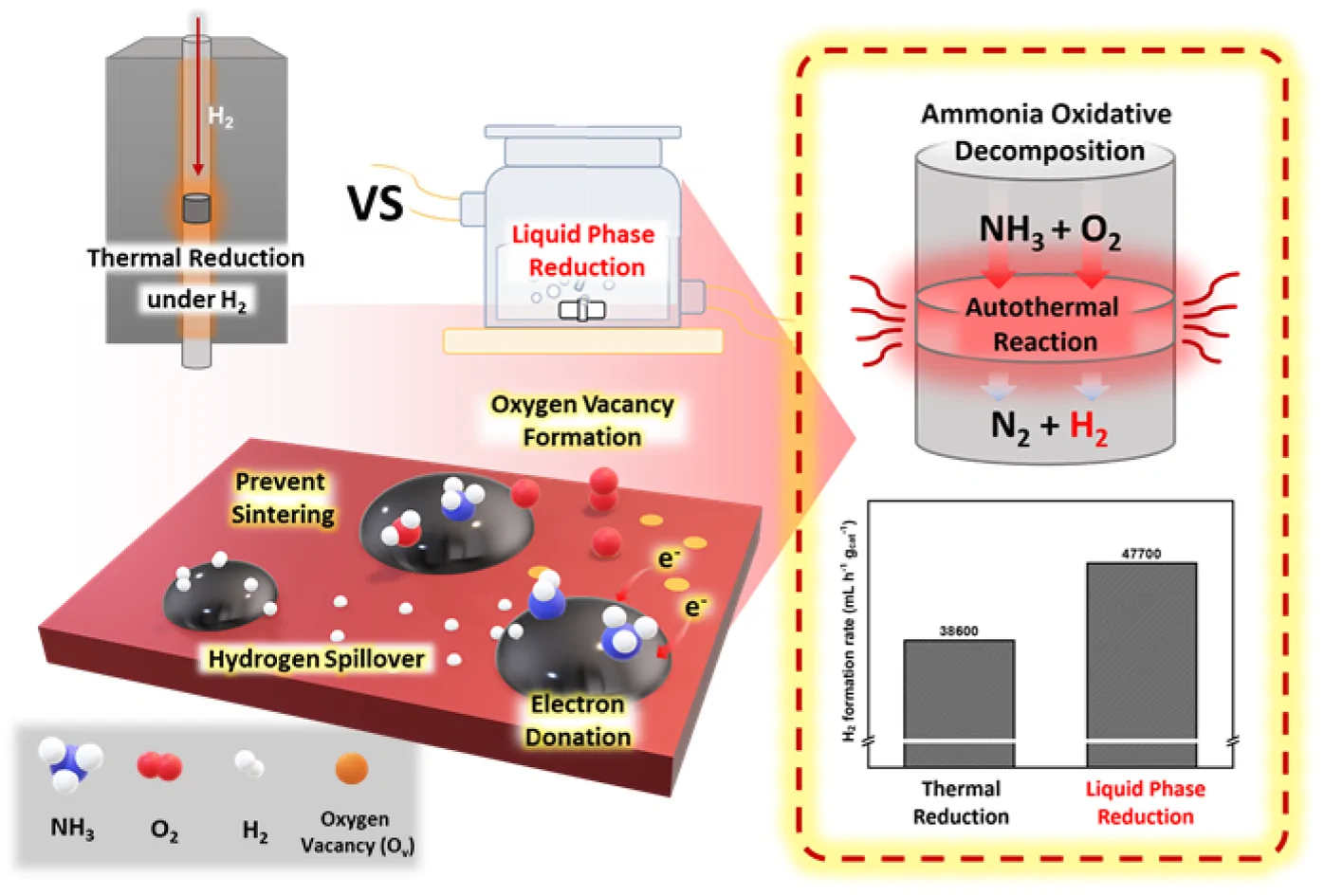

## 1 Introduction

The Paris Agreement (2015) established binding targets for reducing greenhouse gas emissions, driving demand for clean energy alternatives.^1^ Hydrogen (H_2_) is a carbon-free energy carrier with no direct CO_2_ emissions, making it one of the most promising alternatives to fossil fuels.^2,3^ Hydrogen has a gravimetric energy density of 34.0 kcal g^−1^, surpassing petroleum (8.4–10.3 kcal g^−1^) by 3–4-fold and coal (7.8 kcal g^−1^) by approximately 5-fold. This makes it a highly promising component of the next generation energy system.^4^ Yet, the low volumetric energy density of hydrogen necessitates the use of efficient carriers for economical long-term storage and transportation.^5^

Ammonia (NH_3_) is one of the most studied substances as a hydrogen carrier. Ammonia is easily transformed into a liquid state at room temperature under 8 bar pressure, offering ease in both transportation and storage. Additionally, as the second most globally produced chemical (around 200 Mt per year), ammonia offers convenience in production, storage, and transportation by utilizing existing infrastructure for its processing and distribution.^6^ Most notably, ammonia boasts a substantial volumetric hydrogen density (120.3 kgH_2_ m^−3^) compared to other hydrogen storage systems like gaseous H_2_ (36 kgH_2_ m^−3^ at 800 bar), liquid H_2_ (70.9 kgH_2_ m^−3^), and liquid organic hydrogen carriers (47.1 kgH_2_ m^−3^ for MCH). This characteristic renders it the most appealing substance as a hydrogen carrier.^7,8^ Nevertheless, the toxicity of ammonia necessitates specialized control systems for safe handling. The decomposition of ammonia into hydrogen and nitrogen with a stoichiometric ratio of 3 : 1 is an endothermic reaction (Δ*H*° = +92.22 kJ mol^−1^). As the reaction is thermodynamically favored at high temperatures, conversion efficiency increases with temperature. Theoretically, a conversion rate exceeding 99% can be achieved above approximately 400 °C, considering the reaction equilibrium. However, in practical applications, external factors such as mass and heat transfer limitations require the reaction to be conducted under significantly high-temperature conditions.^9–11^ Furthermore, the decomposition process of ammonia requires high temperature for dehydrogenation, which, in turn, demands a substantial energy input to produce hydrogen.^6,12,13^ Consequently, considerable research effort has been devoted to improving the efficiency of ammonia decomposition. Among these strategies, catalyst development has been the most actively pursued approach. As the reverse of the ammonia synthesis reaction, the ammonia decomposition reaction shares essential features with ammonia synthesis, and thus various metal-based catalysts, starting from Fe-based systems to Ru, Ni, Co, Ir, Mo, Pd, Rh, and Cu, have been reported for this reaction.^14^ In particular, Ru-based catalysts have been identified as exhibiting superior catalytic activity under milder conditions compared to other metal-based catalysts. This outstanding performance is attributed to the B5 active sites of Ru, consisting of a layer of three Ru atoms on a single-atom step of the Ru(0001) surface and two Ru atoms directly above it, where ammonia decomposition is known to predominantly occur.^15^ The rational design of Ru-based catalysts has been extensively studied, revealing a size-dependent catalytic behavior, where smaller Ru nanoparticles demonstrate higher activity.^16^ To achieve this, various supports have been explored to utilize strong metal–support interaction (SMSI) effects, while other studies have focused on modifying the chemical state of Ru by introducing promoters.^17^ Moreover, recent studies have reported alloying strategies such as Co–Ni and Ru–Ni alloys to further enhance the catalytic activity for ammonia decomposition.^18^ The central aim of these efforts is to reduce the activation energy of ammonia decomposition and improve H_2_ productivity.^19^

Recently, Nagaoka *et al.* devised a system for the oxidative decomposition of ammonia (ODA). This system involves introducing O_2_ into the ammonia feed stream to simultaneously trigger ammonia decomposition and oxidation reactions.^20,21^ The oxidative decomposition reaction of ammonia occurs through an autothermal process, utilizing the heat generated from the combustion of ammonia to supply the heat required for the endothermic decomposition of ammonia. Nagaoka *et al.* initiated the oxidative decomposition of ammonia without external heat supply using Ru/Al_2_O_3_ and Ru/Ce_0.5_Zr_0.5_O_2−*x*_ catalysts, achieving remarkable NH_3_ conversion rates in this system. Hence, the oxidative decomposition of ammonia can sustain the reaction without continuous external heat supply, unlike ammonia decomposition. Furthermore, the internal heat generation characteristic of ODA mitigates the heat-transfer limitations that are inherent to endothermic ammonia decomposition, particularly at larger scales where maintaining uniform temperature profiles is challenging. However, unlike the 100% theoretical maximum H_2_ yield observed in the ammonia decomposition reaction, the oxidative decomposition of ammonia involves the consumption of a portion of the supplied ammonia for the combustion process, consequently resulting in a reduced theoretical maximum H_2_ yield (66.7%, where NH_3_/O_2_ = 4). The primary focus of this study was to enhance catalyst efficiency by decreasing the proportion of ammonia oxidation, aiming to elevate the theoretical maximum H_2_ yield and maximize H_2_ production even under reduced heat supply.

For ammonia decomposition, Ru, Ir, Ni, Pt, Pd, and Fe have been widely studied as active metals. Specifically, Ru catalysts supported on diverse metal oxides like Al_2_O_3_, MgO, CeO_2_, ZrO_2_, and Y_2_O_3_ have been extensively researched and are known for their superior activity.^22^ Within metal oxide structures, an oxygen vacancy occurs when oxygen atoms are absent, resulting in a structural defect. Within catalysts, oxygen vacancies serve as active sites in oxidation reactions such as ammonia oxidation. In ammonia decomposition reactions, they enhance activity by inducing electron donation, thereby facilitating the rate-determining step of nitrogen recombinative desorption. Additionally, oxygen vacancies trigger the hydrogen spillover effect, preventing hydrogen poisoning of active metals, thereby facilitating the smooth progression of the ammonia decomposition reaction.^23–29^ Oxidative decomposition of ammonia is a complex reaction involving simultaneous oxidation and decomposition processes of ammonia. Therefore, oxygen vacancies within the catalyst could play a crucial role in enhancing the activity of both the oxidation and decomposition reactions of ammonia. To maximize the catalysis for ammonia oxidative decomposition, it's crucial to increase the concentration of oxygen vacancies and determine the optimal concentration required for efficient oxidative decomposition of ammonia.

The creation of oxygen vacancies in metal oxides requires an inherent reduction process. Conventionally, catalysts are reduced through thermal processes under an H_2_ atmosphere. To increase the concentration of oxygen vacancies in the catalyst through thermal reduction, the reduction process needs to occur at temperatures higher than those required for metal reduction. This might lead to the sintering of active metals and an increased demand for energy consumption. Zhang *et al.* previously reported that the concentration of oxygen vacancies in TiO_2_ can be modified *via* liquid-phase reduction utilizing a sodium borohydride (NaBH_4_) solution.^30^ Utilizing NaBH_4_ in liquid-phase reduction enables safe and efficient reduction at approximately room temperature, thereby preventing the sintering of metal particles.

This study focused on enhancing the ammonia oxidative decomposition performance of the Ru/CeO_2_ catalyst, one of the most favorable catalysts for ammonia decomposition, by introducing NaBH_4_ liquid-phase reduction. This approach effectively induced oxygen vacancies, thereby maximizing the catalyst's performance in the oxidative decomposition of ammonia.

## 2 Experimental

### 2.1 Chemical and materials

Ruthenium(iii) nitrosyl nitrate solution (Ru(NO)(NO_3_)_*x*_(OH)_*y*_, *x* + *y* = 3, 1.5 wt%), ethyl alcohol (C_2_H_6_O, pure, ≥99.5%), cerium(iv) oxide (CeO_2_, 99.9%), and sodium borohydride (NaBH_4_, 98%) were purchased from Sigma-Aldrich.

### 2.2 Preparation of catalysts

Ru/CeO_2_ catalyst was synthesized by wet impregnation method, targeting 5 wt% Ru content. Ruthenium(iii) nitrosyl nitrate solution was added into ethyl alcohol under vigorous stirring. Then, cerium(iv) oxide support was added into the above solution and continued stirring for 10 min. The solvent was then removed by using a rotary evaporator. Subsequently, the obtained powder was calcined under an Ar atmosphere at 600 °C for 5 h.

In thermal reduction process, the catalysts were reduced under 10 mol% H_2_/Ar, at 250 °C and 900 °C for 1 h. The obtained catalysts were named LT and HT, respectively. The reduction temperatures were selected based on the H_2_-TPR profile of Ru/CeO_2_ (Fig. S1), which shows a low-temperature reduction region assigned to RuO_*x*_ and surface CeO_2_ and a separate high-temperature region assigned to bulk CeO_2_ reduction.^31^ Accordingly, 250 °C was chosen to reduce RuO_*x*_ and the ceria surface without inducing bulk reduction, whereas 900 °C was chosen to additionally drive bulk CeO_2_ reduction.

In liquid-phase reduction process, NaBH_4_ was added into DI water (18.2 MΩ cm). Then the catalysts were reduced in 3 M NaBH_4_ solution at 10 °C for 12 h under vigorous stirring. After this reduction treatment, the samples were washed with aqueous ethyl alcohol and acetone by centrifugation several times, then dried overnight at 50 °C under reduced pressure. The obtained catalyst was named LPR.

### 2.3 Characterization

To analyze the chemical composition of prepared samples, inductively coupled plasma-optical emission spectrometry (ICP-OES) was performed with Varian 700-ES. Before the measurement, samples were dissolved in a mix of hydrochloric acid (HCl), nitric acid (HNO_3_), and perchloric acid (HClO_4_) at a 6 : 2 : 1 ratio and then diluted with deionized water (H_2_O). The residual sodium and boron contents of the liquid-phase-reduced catalysts and the sodium contents of the Na-impregnated control catalysts were determined by the same procedure, and the results are summarized in Table S2.

X-ray diffraction (XRD) was conducted to assess the crystalline structure of Ru/CeO_2_ catalysts. The diffraction patterns were obtained by a Dmax2500/PC (Rigaku) with Cu Kα source (40 kV, 150 mA) in the 2 theta range of 10–90°.

Transmission electron microscopy (TEM) images with corresponding energy-dispersive X-ray spectroscopy (EDS) were obtained by Tecnai G2 F30ST (FEI) operating at 300 kV to determine Ru metal particle size.

X-ray photoelectron spectroscopy (XPS) was operated on a K-alpha+ (Thermo Fisher Scientific) with Al Kα micro-focused monochromator to analyze the electronic state of the catalysts. The binding energies of XPS spectra were corrected to the C 1s binding energy at 284.8 eV.

H_2_-temperature programmed reduction (H_2_-TPR) was carried out with an AutoChem II 2920 (Micromeritics) to determine the reducibility of the catalysts. Samples were pretreated under flowing He at room temperature to remove surface-adsorbed impurities, and they were heated in the temperature range 50–900 °C at a ramping rate of 10 °C min^−1^ under 10 mol% H_2_/Ar. H_2_ consumption was monitored by a thermal conductivity detector (TCD).

CO and H_2_ pulse chemisorption were performed on an AutoChem II 2920 (Micromeritics) to quantify the exposed Ru sites and to estimate the extent of hydrogen spillover. On ceria-supported metals, CO reacts with the lattice oxygen of CeO_2_ to form carbonates, so that direct CO pulsing overestimates both the CO uptake and the derived metal dispersion. To suppress this contribution, the O_2_–CO_2_–H_2_–CO pretreatment established for CeO_2_-containing supports was adopted.^32,33^

The sample was first exposed to 5 mol% O_2_/He at 300 °C for 10 min, cooled to 50 °C under He, reduced under 5 mol% H_2_/Ar at 250 °C for 15 min, and cooled again to 50 °C. It was then treated at 50 °C in the following sequence. He was flowed for 5 min, followed by 5 mol% O_2_/He for 5 min, which re-oxidizes the reduced ceria surface and restores the surface oxygen required for carbonate formation. The sample was subsequently held under 10 mol% CO_2_/He for 10 min to saturate the ceria surface with carbonates, so that no further carbonate forms during the following CO pulses and only metallic Ru remains available for CO adsorption; this blocking effect has been verified by IR and DRIFTS.^32,33^ He was then flowed for 20 min to remove weakly bound CO_2_, and finally 5 mol% H_2_/Ar was introduced for 5 min to reduce the Ru surface oxidized during the preceding O_2_ step back to the metallic state. Because this reduction is carried out at 50 °C, the carbonates formed on the support are retained and the blocking effect is preserved. After a He purge, 10 mol% CO/He pulses were introduced at 50 °C until the CO uptake was saturated, and the uptake was measured with a TCD.

For H_2_ chemisorption, the sample was pretreated under heat 150 °C for 1.5 h, cooled to 50 °C, and 5 mol% H_2_/Ar was pulsed repeatedly until saturation. The surface concentrations of adsorbed CO and H were determined assuming CO : Ru = 1 : 1 and H_2_ : Ru = 1 : 2, and the extent of hydrogen spillover was evaluated as *Q*_spillover_ = 2*Q*_H_2__ − *Q*_CO_. Because a contribution from hydrogen retained on Ru after the final reduction step cannot be entirely excluded, *Q*_spillover_ is used here as a comparative measure among the three catalysts rather than as an absolute quantification.

Temperature-programmed desorption (TPD) was carried out on a BELCAT-B (BEL Japan) with TCD. Pretreatment was conducted by following this procedure. For H_2_-TPD, catalyst was exposed under He at 150 °C for 1.5 h, and then exposed to 10 mol% H_2_/Ar at 100 °C for 1 h. Finally, the catalyst was purged under He for 30 min. For N_2_-TPD analysis, catalyst was pretreated under He at 400 °C for 1.5 h. Next, N_2_ was adsorbed onto the catalyst at 400 °C for 1 h. After cooling to 35 °C under N_2_, the catalyst was purged under He for 30 min. After pretreatment, desorption was conducted under He at the temperature from 35 °C to 850 °C at a ramping rate of 10 °C min^−1^ during all of the TPD analysis.

To examine whether nitrogen oxides are formed as a function of the feed composition, a separate set of experiments was carried out using a commercial 5 wt% Ru/Al_2_O_3_ catalyst (Sigma-Aldrich) while varying the NH_3_ : O_2_ ratio. FT-IR absorbance spectra of the effluent were recorded on a Nicolet iS50 spectrometer (ThermoFisher Scientific) equipped with a gas cell. The gas cell was placed downstream of the reactor and the cold trap but upstream of the H_2_SO_4_ and DI water traps, so that the effluent was analyzed before passing through the traps; the gas stream then flowed through the traps to the GC.

Electron energy loss spectroscopy (EELS) of the Ce M_4,5_ edge was performed by field-emission transmission electron microscopy (FE-TEM; Libra 200 MC, Carl Zeiss; AE12) at an accelerating voltage of 200 kV at the Korea Basic Science Institute (KBSI), Daejeon, Korea. The Ce M_4,5_-edge EELS spectra were acquired with an energy dispersion of 0.25 eV per channel to investigate the local electronic structure and coordination environment of Ce species. The pre-edge background was subtracted, and any residual offset was removed by subtracting the mean intensity over 866–876 eV and the energy axis was calibrated by setting the CeO_2_ M_5_ maximum to 883.3 eV. For quantitative comparison, the integrated white-line intensity ratio *I*_M_5__/*I*_M_4__ was determined by integrating the M_5_ white line over 876–893 eV and the M_4_ white line over 893–912 eV, the upper limit of the M_5_ window being placed at the minimum between the two white lines. The same integration limits were applied to all three catalysts. This procedure follows methods outlined in the literature.^34,35^

### 2.4 Catalytic performance measurements

The catalyst test was conducted for two reactions: oxidative decomposition and decomposition reaction of ammonia. Both reactions were conducted in a quartz tube fixed-bed reactor with an inner diameter of 8 mm.

For ammonia oxidative decomposition reaction, 80 ml min^−1^ of pure gaseous NH_3_, 20 ml min^−1^ of O_2_, and 5 ml min^−1^ of He were fed into 50 mg of catalyst (GHSV = 126 000 ml h^−1^ g_cat_^−1^) under ambient pressure for 3 hours reaction. The reactant gases were supplied at room temperature external heating consisted solely of a temperature-programmed ramp at 20 °C min^−1^ to a set point of 300 °C, which was completed within *ca.* 15 min of start-up. The furnace was then switched off, and no external heat was supplied for the remainder of the 3 h run (Fig. S2). A K-type thermocouple was positioned 1 mm upstream of the catalyst bed and was used both to record the bed temperature and to control the furnace during the ignition step; because the catalyst bed is only *ca.* 2 mm thick, a thermocouple could not be inserted within or downstream of the bed without disturbing its structure. The effluent was passed through a cold trap held at 10 °C to remove the water formed by ammonia combustion before analysis.

For ammonia decomposition reaction, 50 ml min^−1^ of pure gaseous NH_3_ and 3 ml min^−1^ of He were fed into 100 mg (GHSV = 31 800 ml h^−1^ g_cat_^−1^) under ambient pressure condition at different temperatures of 400, 500, 600, and 700 °C each for 2 hours.

Unreacted NH_3_ in the effluent gases was removed in trap of H_2_SO_4_ and DI water, and H_2_O produced by the reaction was removed in cold trap. Then, the remaining reaction products of reaction (H_2_ and N_2_) and inert He were analyzed by gas chromatography (YL 6500, Young In Chromass) with a molesieve 5 Å column and TCD. Helium served as an internal standard for calculations of NH_3_ conversions and H_2_ yields, and the conversion and yield were calculated based on following equations:NH_3_ → 1/2N_2_ + 3/2H_2_
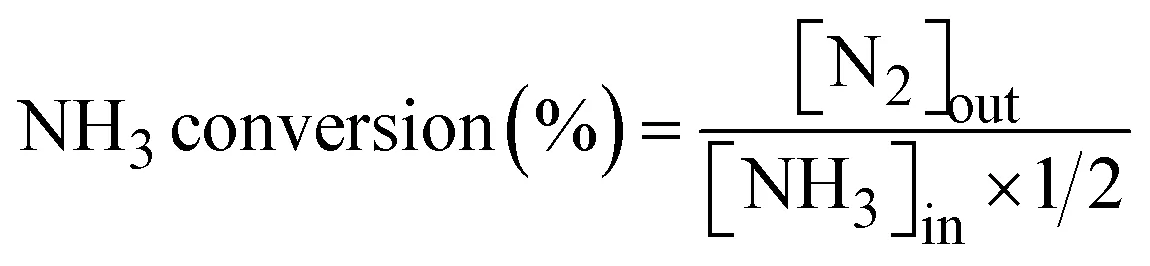

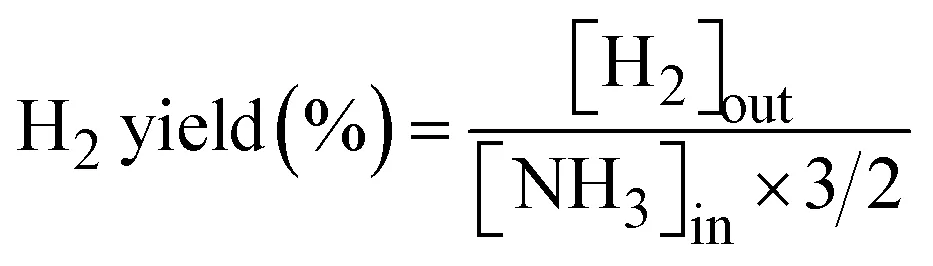

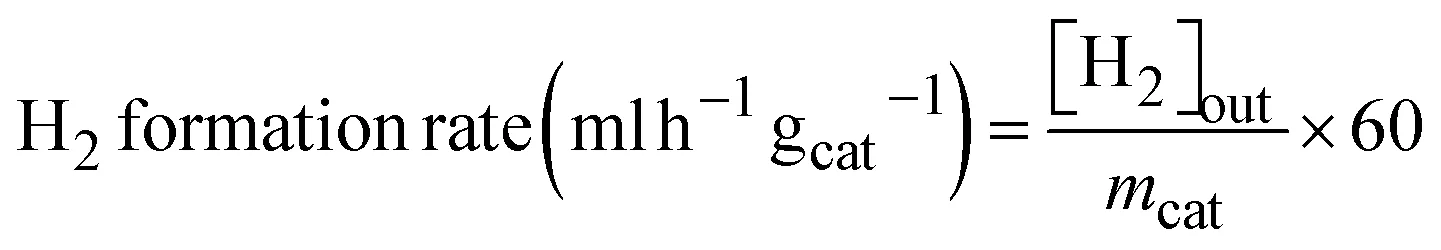
where [NH_3_]_in_, [N_2_]_out_, [H_2_]_out_, *m*_cat_ are the volumetric flow of NH_3_ in reactant flow, the volumetric flow of N_2_ in exit flow, the volumetric flow of H_2_ in exit flow and the mass of catalyst, respectively.

### 2.5 Thermodynamic and techno-economic evaluation

The autothermal oxidative decomposition of ammonia (ODA) was evaluated using a unified framework combining standard-state thermochemical analysis and a concept-level cost model. Standard-state thermodynamic data for NH_3_, N_2_, H_2_, O_2_, and H_2_O were retrieved from the NIST Chemistry WebBook, and no electronic-structure calculations were performed. Reaction enthalpies were derived using Hess's law for the decomposition and oxidation reactions:
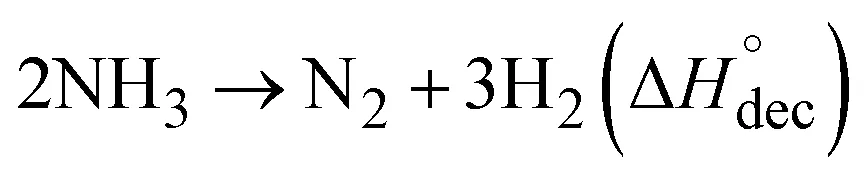

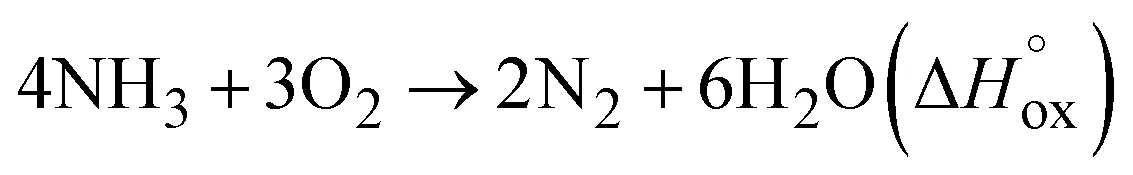


The enthalpies normalized per mole of NH_3_ were calculated as 
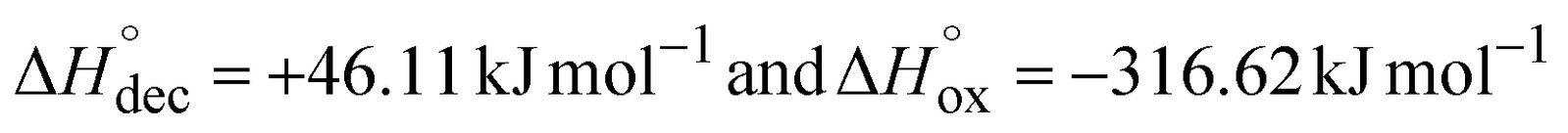
. Gibbs free energy profiles Δ*G*^°^(*T*) were generated over the temperature range of 298–1200 K to confirm that decomposition becomes spontaneous above approximately 460 K, while oxidation remains thermodynamically favorable throughout this range.

To determine the condition for autothermal operation, the adiabatic energy balance was solved using the following equation:*α*Δ*H*_ox_/NH_3_ + (1 − *α*)(Δ*H*_dec_/NH_3_) = 0where *α* represents the fraction of ammonia oxidized. The theoretical thermoneutral point was determined to be *α* ≒ 0.127. Under the experimental conditions employed in this study (NH_3_/O_2_ = 4, corresponding to an O_2_/NH_3_ ratio of 0.25), the oxidation fraction (*α*) is fixed at 1/3 (0.333). This results in a net heat release of approximately 75 kJ mol^−1^ NH_3_ (Δ*H* = −75 kJ mol^−1^ NH_3_), enabling self-sustained ignition near 455 K. Consequently, the theoretical maximum hydrogen yield is limited to 66.7%, as only the non-oxidized fraction (1 − *α*) of ammonia undergoes decomposition.

For the economic assessment, the Levelized Cost of Hydrogen (LCOH) was estimated utilizing a bottom-up model that accounts for feedstock (ammonia), oxygen, catalyst replacement, electricity, and capital recovery. The base-case parameters were defined as follows: ammonia price of $400 per ton; oxygen price of $50 per ton (assuming supply from a small-scale PSA unit); electricity cost of $0.08 kWh^−1^ with a consumption of 2.5 kWh kg-H_2_^−1^. The catalyst was assumed to be 5 wt% Ru/CeO_2_ with a 3 year lifetime and a productivity of 3.3 kg-H_2_ kg-cat^−1^ day^−1^. The installed capital expenditure (CAPEX) was estimated at $500 per kg-H_2_ day^−1^ with a project lifespan of 15 years and a weighted average cost of capital (WACC) of 8%. The single-pass hydrogen yield of the base case was set to the experimental value of the LPR catalyst (26.4%, *i.e.*, 40% of the 66.7% ceiling), and a yield of 33% (50% of the ceiling) was evaluated as an improvement scenario.

## 3 Results and discussion

### 3.1 Catalytic activities

Fig. 1a illustrates the NH_3_ conversion and H_2_ yield in oxidative decomposition of ammonia for three reduced catalysts, while Fig. 1b displays the results of NH_3_ conversions in the decomposition of ammonia reaction. In Fig. 1a, the NH_3_ conversion during the NH_3_ oxidative decomposition reaction, where oxidation and decomposition occur simultaneously, is observed at 35.3% for the HT catalyst, 54.5% for the LT catalyst, and 60.3% for the LPR catalyst. Additionally, the HT, LT, and LPR catalysts respectively exhibit hydrogen yields of 3.68%, 20.6%, and 26.4%. The conversion of NH_3_ and the yield of H_2_ were calculated based on the concentrations of N_2_ and H_2_ observed in gas chromatography. Nitrogen is generated from both the oxidation and decomposition reactions of ammonia, while hydrogen is primarily produced from the decomposition of ammonia. Therefore, the conversion of NH_3_ reflects the combined extent of both the oxidation and decomposition reactions of ammonia, whereas the H_2_ yield signifies the degree of ammonia decomposition. As shown in Fig. S3, no O_2_ peak was detected in the GC data. This absence indicates the complete consumption of the introduced O_2_ during the oxidative decomposition of ammonia. Since the H_2_ yield reflects the decomposition reaction alone, the extent of the oxidation reaction can be estimated by subtracting the H_2_ yield from the NH_3_ conversion. All the prepared catalysts exhibited a consistent gap of approximately 32–34% between the H_2_ yield and the NH_3_ conversion, suggesting that the supplied O_2_ was predominantly consumed through the complete oxidation of NH_3_ (theoretical maximum conversion *via* NH_3_ oxidation: 33%). This value is largely fixed by the oxygen balance once the oxygen is fully consumed, and its agreement with experiment therefore serves as a consistency check on the oxygen and nitrogen balances rather than as an independent measure of catalytic behavior. The catalyst bed temperature offers additional evidence for this interpretation. Given that the extent of ammonia oxidation is essentially identical across the three catalysts, the heat released by oxidation is also fixed, and the bed temperature is therefore governed by how much of that heat is consumed by the endothermic decomposition reaction. As shown in Fig. 1a, the bed temperature decreases in the order HT (534.7 °C) > LT (470.8 °C) > LPR (248.4 °C), the inverse of the order of H_2_ yield. Accordingly, it can be inferred that the observed differences in NH_3_ conversion were predominantly governed by the extent of the ammonia decomposition reaction. The autothermal character of the operation is further illustrated by the time-dependent temperature profile recorded over the LPR catalyst (Fig. S2). External heating consisted solely of the 20 °C min^−1^ ramp to the 300 °C set point, which was completed within *ca.* 15 min of start-up; the furnace was then switched off and delivered no power for the remaining 3 h of the run. Ignition occurred during this ramp, at approximately 455 K (*ca.* 182 °C) and thus well below the set point, as evidenced by a sharp rise of the bed temperature above the furnace ramp. After the furnace was switched off, the bed temperature continued to rise, reaching *ca.* 320 °C, which is above the furnace set point, before relaxing to a steady value of *ca.* 250 °C, in agreement with the steady bed temperature of the LPR catalyst in Fig. 1a; this temperature was then maintained for the remainder of the run without any external heat input. This behavior directly demonstrates the operating concept of ODA: the heat released by the exothermic combustion of part of the ammonia feed sustains the catalyst bed temperature, and this heat in turn drives the endothermic decomposition of the remaining ammonia. Ammonia oxidation can in principle generate nitrogen oxides through incomplete oxidation. To examine this possibility, the reactor effluent was analyzed by FT-IR over a wide range of feed compositions using a commercial 5 wt% Ru/Al_2_O_3_ catalyst (Fig. S4); the gas cell was located downstream of the cold trap but upstream of the acid traps, so that the spectra reflect the product gas before any nitrogen-containing species could be removed (Section 2.3). At the feed composition employed throughout this study (NH_3_ : O_2_ = 80 : 20, O_2_/NH_3_ = 0.25), only absorptions of residual NH_3_ were observed, and the spectrum remained essentially unchanged up to NH_3_ : O_2_ = 80 : 50 (O_2_/NH_3_ = 0.625). Nitrogen oxides emerged only when the feed ratio reached O_2_/NH_3_ = 0.75: NO absorptions first appeared at this ratio, and N_2_O became evident as the ratio approached 1.0. These onsets reflect a single stoichiometric threshold. Complete oxidation of NH_3_ to N_2_ (4NH_3_ + 3O_2_ → 2N_2_ + 6H_2_O) requires O_2_/NH_3_ = 0.75; below this ratio the oxygen feed is sub-stoichiometric even for complete combustion to N_2_, so the supplied O_2_ is consumed essentially completely, the steady-state coverage of adsorbed oxygen remains low, and surface nitrogen is removed by recombination to N_2_. Once the feed reaches this ratio, the oxygen supply is no longer sub-stoichiometric with respect to N_2_ formation: surplus adsorbed oxygen becomes available on the surface, the pathway coupling surface N and O atoms becomes accessible, and NO emerges at the threshold itself, followed by N_2_O at higher oxygen ratios. With increasing O_2_ ratio, the NO and N_2_O bands intensified, the residual NH_3_ bands were progressively attenuated as a larger fraction of the feed was combusted, and H_2_O bands began to appear because the larger amount of water formed was no longer completely removed by the cold trap. The feed composition of this study, O_2_/NH_3_ = 0.25, corresponds to only one third of the ratio at which nitrogen oxides begin to form, placing the ODA operation deep within the NO_*x*_-free regime. This threshold behavior is consistent with reports that the selectivity of NH_3_ oxidation is governed by the surface oxygen coverage, NO formation being favored at high adsorbed O/NH_*x*_ ratios;^36,37^ under oxygen-limited conditions the surface nitrogen is instead removed by recombination to N_2_. In addition, since only about one third of the supplied NH_3_ is consumed by oxidation, the large excess of unreacted NH_3_ together with the H_2_ produced maintains a reducing environment in which any nitrogen oxides formed over Ru are reduced to N_2_.^38,39^ Because the onset of NO_*x*_ formation is dictated by the oxygen stoichiometry of the feed rather than by the identity of the catalyst, all catalysts operated at O_2_/NH_3_ = 0.25 are expected to remain essentially NO_*x*_-free. This expectation is corroborated by the conversion-yield gap of approximately 33% noted above, which coincides with the maximum combustion fraction (33.3%) imposed by the oxygen feed. Therefore, the H_2_ yield in ODA is primarily governed by the ammonia decomposition activity of the catalyst. To verify this, an ammonia decomposition (AD) reaction was conducted without any oxidant.

**Fig. 1:**
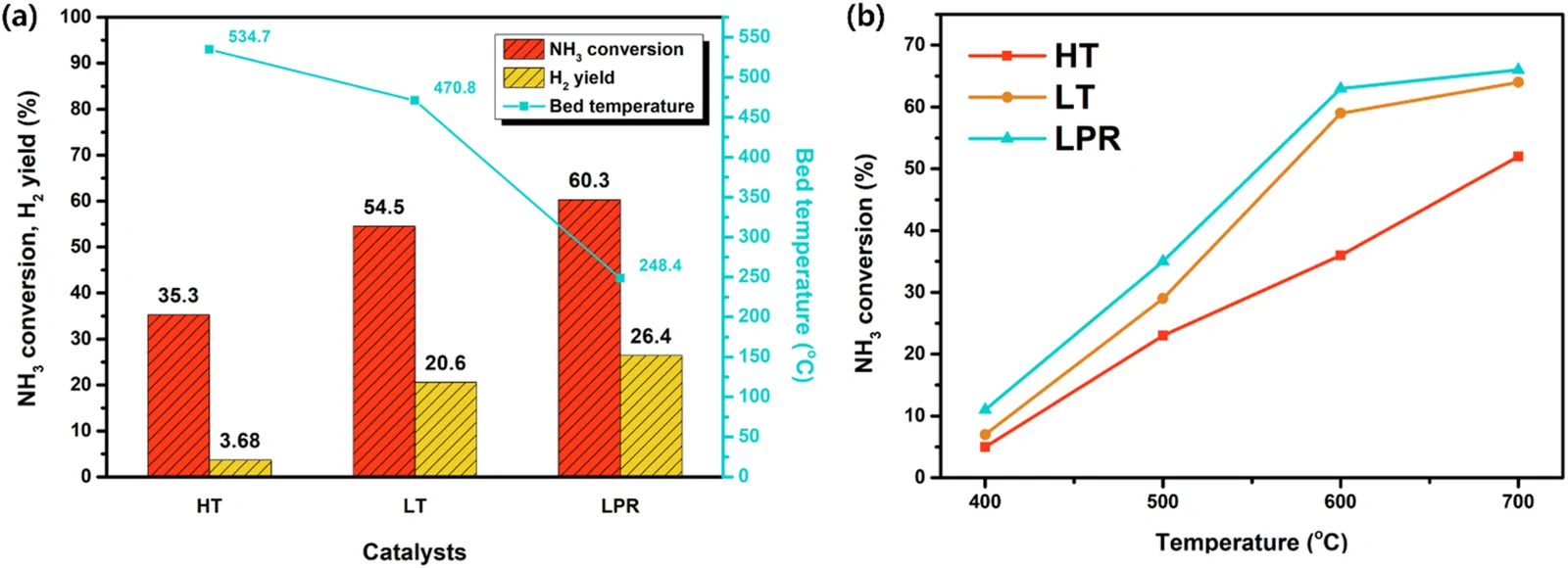
(a) Catalytic activities and bed temperature of Ru/CeO_2_ catalysts for oxidative decomposition of ammonia at feed gas composition of NH_3_ : O_2_ : He = 80 : 20 : 5 ml min^−1^ (GHSV = 126 000 ml h^−1^ g_cat_^−1^), (b) NH_3_ conversions of Ru/CeO_2_ catalysts for decomposition of ammonia at feed gas composition of NH_3_ : He = 50 : 3 ml min^−1^ (GHSV = 31 800 ml h^−1^ g_cat_^−1^).

Furthermore, in Fig. 1b, conversions of NH_3_ at different temperatures were depicted for the ammonia decomposition reaction. Over a range of temperatures, each catalyst exhibits a consistent trend in activity. Specifically, at all temperatures, LPR catalyst exhibits the highest ammonia conversion, followed by LT and HT catalysts in their respective activity levels. Especially in the case of the HT catalyst, a significantly low activity in ammonia decomposition can be observed. Comparison of Fig. 1a and b reveals that across both the oxidative decomposition and ammonia decomposition reactions, LPR, LT, and HT showed a consistent order of reaction activity. This observation implies a correlation between the H_2_ yield in the oxidative decomposition reaction and the trend observed in the decomposition reaction.

### 3.2 Structural properties of catalysts

To elucidate the differences in activity among these catalysts, comprehensive characterization analysis of each catalyst was conducted. First, X-ray diffraction (XRD) and transmission electron microscopy (TEM) analysis were conducted for structural characterization of the catalyst.

In the XRD pattern (Fig. 2a), the crystal structure of CeO_2_ is evident at 28.5°, 33.0°, 47.4°, 56.3°, 59.0°, 69.3°, 76.6°, 79.0°, and 88.4° (JCPDS 43-1002). The absence of any peak shifts across all catalysts consistently validates the unchanged state of the CeO_2_ bulk structure during the catalyst pre-treatment. On the other hand, distinct difference was apparent in the ruthenium peak within the entire diffraction pattern. While no diffraction peaks other than those of CeO_2_ were detected in the LT and LPR catalysts, a small peak related to metallic Ru was detected at 44.0° (JCPDS 06-0663) in the HT catalyst. This implies the presence of large metallic ruthenium crystallites in the HT catalyst. Considering that the HT catalyst underwent an intense reduction process at approximately 900 °C, its exposure to such high temperatures may have caused the sintering of ruthenium.

**Fig. 2:**
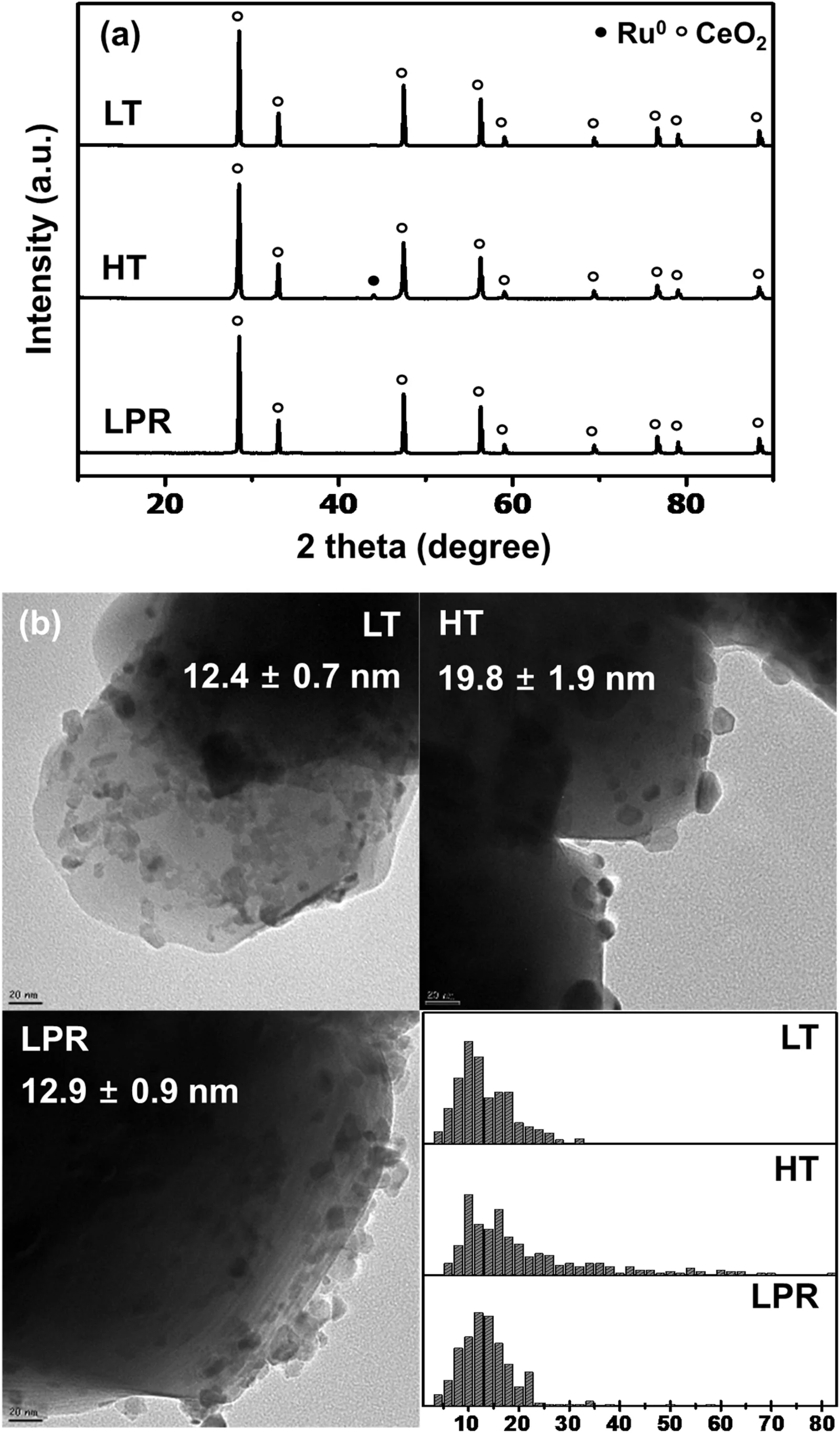
(a) XRD patterns, and (b) TEM images and Ru size distribution of the Ru/CeO_2_ catalysts.

This sintering tendency is also evident in the TEM images (Fig. 2b). The Ru particle sizes observed in Fig. 2b were relatively similar between the LT and LPR catalysts, measuring 12.4 ± 0.7 nm and 12.9 ± 0.9 nm, respectively. However, the HT catalyst exhibited a larger Ru particle size, measuring 19.8 ± 1.9 nm. Consistent with the trends seen in the XRD patterns, high-temperature reduction of the catalyst leads to undesirable sintering of Ru and ultimately reduces catalyst activity.

### 3.3 Electronic state of catalysts

Following the structural analysis of the catalysts, X-ray photoelectron spectroscopy (XPS) analysis was conducted to examine the generation of oxygen vacancies and the electronic state of ruthenium (depicted in Fig. 3 and Table 1). Fig. 3a shows the narrow-scan XPS spectrum of the O 1s region of each catalyst. The O 1s spectrum is deconvoluted into three components: O_α_ (∼529.5 eV) representing lattice oxygen existing in CeO_2_ crystals, O_β_ (∼531.1 eV) indicating oxygen vacancy, and O_γ_ (∼532.6 eV) assigned to adsorbed water and weakly bound surface species. The O_β_/O_α_ ratio amounts to 0.57 for LT, 0.29 for HT and 1.23 for LPR (Table 1). Although this ratio has been used as an indicator of oxygen vacancies,^40,41^ the O_β_ component cannot be separated from surface hydroxyl, carbonate and adsorbed oxygen species, and the ordering of the three catalysts also follows that of their final treatment temperature (50 °C for LPR, 250 °C for LT, 900 °C for HT), which governs the residual hydroxyl and carbonate coverage. The O_β_/O_α_ ratio is therefore reported only as an upper bound on the vacancy-related contribution and is not used to rank the vacancy concentration; the ranking is based on the Ce 3d spectra and on the Ce M_4,5_-edge EELS measurement described below.

**Fig. 3:**
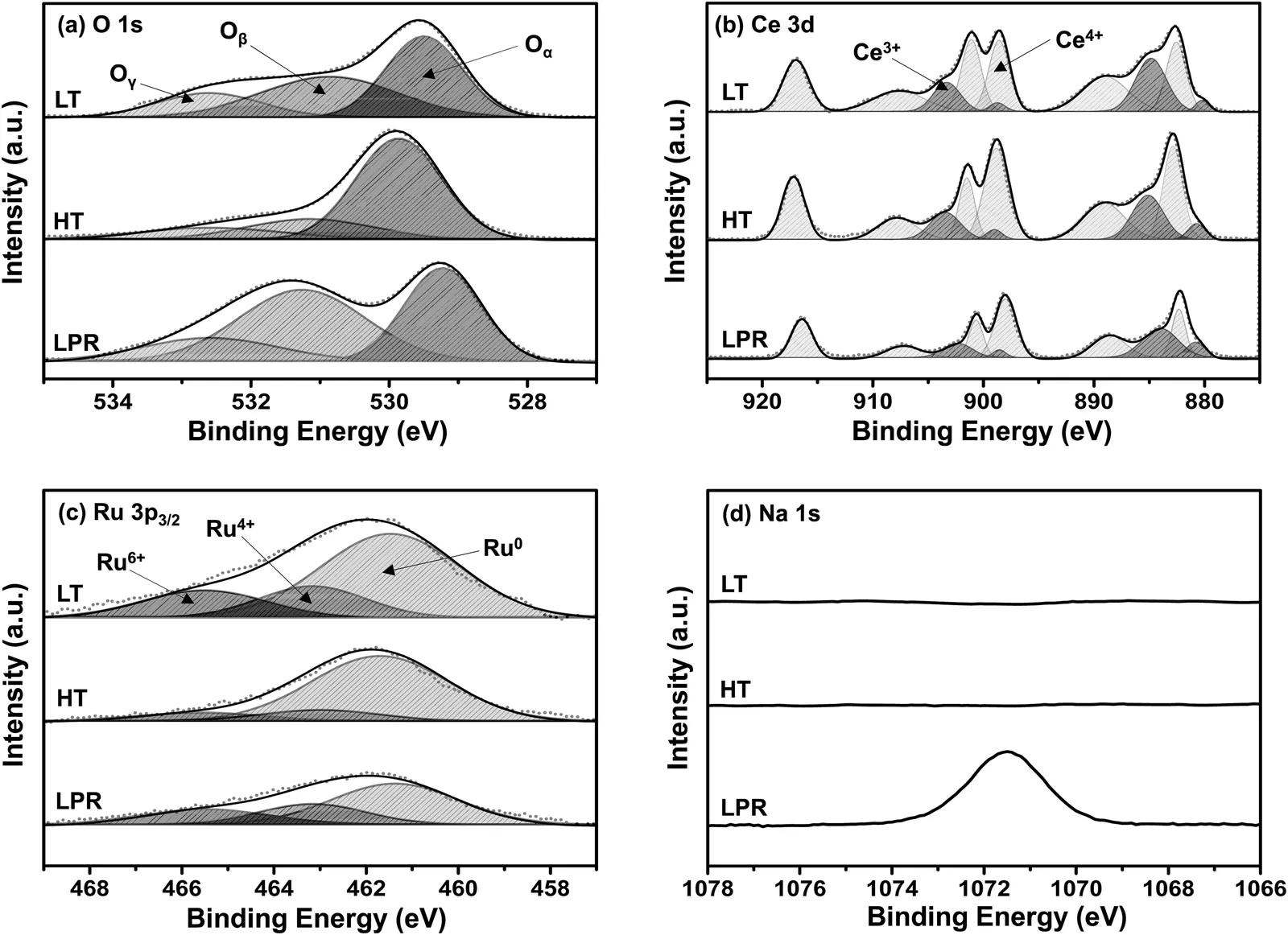
XPS analysis data of Ru/CeO_2_ catalysts – (a) O 1s, (b) Ce 3d, (c) Ru 3p_3/2_, (d) Na 1s.

**Table 1:** The surface analysis from XPS data of Ru/CeO_2_ catalysts^a^

Catalyst	O 1s (%)				Ce 3d (%)			Ru 3p_3/2_ (%)		
	O_α_	O_β_^b^	O_γ_	O_β_/O_α_	Ce^3+^	Ce^4+^	Ce^3+^/Ce^4+^	Ru^0^	Ru^4+^	Ru^6+^
LT	51.1 (529.5 eV)	29.2 (531.1 eV)	19.6 (532.6 eV)	0.57	24.5	75.5	0.32	63.5 (461.4 eV)	18.0 (463.1 eV)	18.4 (465.5 eV)
HT	67.7 (529.8 eV)	19.9 (531.1 eV)	13.8 (532.6 eV)	0.29	22.7	77.3	0.29	79.1 (461.7 eV)	11.5 (463.1 eV)	9.5 (465.6 eV)
LPR	38.0 (529.2 eV)	46.8 (531.2 eV)	15.1 (532.6 eV)	1.23	28.7	71.3	0.40	63.8 (461.3 eV)	18.6 (463.1 eV)	17.6 (465.4 eV)

a Binding energies referenced to adventitious C 1s at 284.8 eV; small sample-to-sample offsets are attributable to the differing surface potentials of the differently reduced samples, and no chemical interpretation is attached to absolute peak positions.

b The O_β_ component includes contributions from surface hydroxyl, carbonate and adsorbed oxygen species; the O_β_/O_α_ ratio therefore represents an upper bound on the vacancy-related contribution.

Additionally, Fig. 3b shows the narrow-scan XPS spectrum of the Ce 3d region. The Ce 3d spectrum consists of four Ce^3+^ peaks and six Ce^4+^ peaks. Similar to O_β_/O_α_, the ratio of Ce^3+^/Ce^4+^ serves as a criterion to verify the abundance of oxygen vacancies.^41,42^ The increasing ratio of Ce^3+^/Ce^4+^ observed in the XPS analysis of the Ce 3d orbital indicates a higher generation of oxygen vacancies in the sequence of LPR > LT > HT. Taken together, Fig. 2, 3b and S5 demonstrate that the liquid-phase reduction method using NaBH_4_ effectively generates oxygen vacancies without inducing Ru sintering. This contrasts with the behavior under H_2_ thermal reduction, where vacancy formation is limited by sintering. Another notable observation is that despite the high-temperature reduction employed for extensive oxygen vacancy formation, the HT catalyst exhibited decreased oxygen vacancies compared to the LT catalyst. This behavior is more plausibly related to the thermal history of the support than to the Ru particles. Treatment at 900 °C is expected to promote sintering and crystallization of CeO_2_ itself, annealing surface defects and reducing the surface area available to host oxygen vacancies, with partial re-oxidation during cooling as a further possible contribution. We note that this interpretation is not established by direct structural evidence in the present work. Sintering of Ru, by contrast, reduces the Ru–CeO_2_ interfacial area and therefore diminishes both Ru–O–Ce linkages and Ruvacancy contacts, consistent with the Ru 3p_3/2_ results discussed below.

Because both the O 1s and Ce 3d analyses rely on peak deconvolution, and because the O_β_ component cannot be separated from surface hydroxyl, carbonate and adsorbed oxygen contributions, the vacancy ordering was further examined by a measurement that does not involve any oxygen-derived signal. Fig. S5 shows the Ce M_4,5_ EELS spectra of the three catalysts, with the M_5_ and M_4_ white lines observed at approximately 883 and 900 eV, consistent with CeO_2_. These white lines arise from Ce 3d to 4f transitions and therefore reflect the occupancy of the Ce 4f orbital, so that a higher *I*_M_5__/*I*_M_4__ ratio indicates a greater abundance of Ce^3+^.^43–45^ Integrating the M_5_ white line over 876–893 eV and the M_4_ white line over 893–912 eV gave a ratio of 0.786 for LPR, compared with 0.708 for LT and 0.710 for HT (Table S1). This difference originates specifically from the M_5_ white line: relative to LT, the integrated M_5_ intensity of LPR is 22% higher whereas the M_4_ intensity increases by only 10%, whereas LT and HT differ by less than 4% in both white lines and therefore give the same ratio. Consistently, LPR exhibits additional intensity on the low-energy-loss side of the M_5_ white line, the fine-structure signature of Ce^3+^; the M_5_ maximum and the high-energy-loss flank of LPR coincide with those of LT while only the low-energy-loss flank is broadened, which excludes a rigid energy shift or a symmetric loss of energy resolution as the origin. Because the Ce M_4,5_ edge probes the cation directly and is unaffected by hydroxyl, carbonate or adsorbed-oxygen species, this result supports the higher oxygen-vacancy concentration of LPR inferred from the O 1s and Ce 3d spectra. The comparable ratios of LT and HT are consistent with the interpretation given above: a loss of vacancies confined to the ceria surface is registered by the surface-sensitive XPS measurement but is diluted in the thickness-integrated EELS signal.

Fig. 3c depicts the results obtained from the narrow-spectrum XPS analysis of the Ru 3p_3/2_ orbital. The Ru 3p_3/2_ orbital comprises three peaks appearing around ∼461.5 eV, ∼463.1 eV, and ∼465.5 eV, indicating Ru^0^, Ru^4+^, and Ru^6+^ state, respectively.^46^ The proportion of metallic Ru, notably higher in HT (79.1%) than LT (63.5%) and LPR (63.8%), suggests a diminished interface between Ru and CeO_2_ in the HT catalyst, possibly due to sintering of Ru, leading to fewer Ru–O–Ce bond formation at the Ru–CeO_2_ interface. The binding energies of metallic Ru are 461.4 eV (LT), 461.7 eV (HT) and 461.3 eV (LPR) (Table 1). These values differ by at most 0.4 eV and follow the same ordering as the O_α_ binding energies, which is characteristic of sample-to-sample offsets in the surface potential of differently reduced ceria rather than of an element-specific chemical shift (footnote to Table 1); after correction for this common offset, the residual differences are ≤0.2 eV and cannot be resolved reliably. We therefore do not use the Ru^0^ binding energy as a measure of the electron density of Ru. Oxygen vacancies of reducible supports are known to donate electron density to the supported metal,^47,48^ and the functional consequence expected from such donation, a weaker Ru–N bond, is examined directly by N_2_-TPD in Section 3.4. That comparison is not confounded by particle-size effects: LT and LPR possess statistically identical Ru particle sizes (12.4 ± 0.7 and 12.9 ± 0.9 nm, Fig. 2b) and closely similar metallic Ru fractions (63.5 and 63.8%), so differences in their N_2_ desorption behavior are attributable to the state of the support rather than to the Ru particles themselves.

Furthermore, Fig. 3d displays the narrow spectrum XPS analysis findings of the Na 1s orbital, conducted to detect any remaining sodium on the catalyst during the NaBH_4_ liquid-phase reduction process. As expected, there were no Na-related peaks observed in LT and HT, whereas a minor peak centered at approximately 1071.3 eV, characteristic of ionic Na^+^, was detected in the LPR catalyst, indicating trace amounts of Na residue (0.02 wt% by ICP-OES, Table S2) remaining on the catalyst after the liquid-phase reduction process. Sodium, as an alkali metal, has been introduced in numerous studies as a promoter for catalysts due to its electron-donating effect, similar to that of oxygen vacancies.^48,49^ The trace sodium retained in the LPR catalyst may therefore act as a mild co-promoter alongside the oxygen vacancies. Since electron donation to Ru is expected to weaken the Ru–N bond and thereby facilitate N_2_ recombinative desorption, the rate-determining step of ammonia decomposition, this contribution would act in the same direction as that of the oxygen vacancies; the N_2_ desorption behaviour of the three catalysts is examined directly in Section 3.4.^25,41,49^

To relate these structural and electronic features to the catalytic behavior, and to isolate the individual contributions of oxygen vacancies, residual sodium and the reduction conditions, a series of control catalysts was evaluated under identical ODA conditions (Fig. S6 and Table S2). First, the three Ru/CeO_2_ catalysts were compared with a commercial 5 wt% Ru/Al_2_O_3_ catalyst (NH_3_ conversion of 48.7% and H_2_ yield of 15.4%). Both LT (54.5%, 20.6%) and LPR (60.3%, 26.4%) clearly outperform the commercial catalyst, whereas HT (35.3%, 3.68%) falls well below it. Although HT possesses oxygen vacancies and the reducible CeO_2_ support that the Al_2_O_3_-supported catalyst lacks, its inferior activity indicates that the severe Ru sintering induced by the 900 °C reduction outweighs these promotional factors and dominates its catalytic behavior. Second, to verify the promoting role of sodium suggested by the Na 1s spectra, Na was deliberately impregnated onto the LT catalyst at nominal loadings of 1 and 5 wt% (measured contents in Table S2). The 1 wt% loading (1Na/LT) increased both the NH_3_ conversion and the H_2_ yield relative to LT (57.7% and 24.0%), consistent with the electron-donating effect of Na, whereas the 5 wt% loading (5Na/LT, 50.2% and 17.2%) decreased the activity below that of unpromoted LT, presumably because the excess Na covers the Ru surface and blocks the active sites. The Na-loading dependence thus captures both the promoting and the inhibiting effects of sodium; in this respect, the trace Na retained in LPR after washing (0.02 wt%, Table S2) lies far below the inhibitory regime and can act only as a mild promoter, in line with the interpretation of Fig. 3d. Finally, the influence of the NaBH_4_ concentration used in the liquid-phase reduction was examined by preparing LPR catalysts in 1, 3 and 5 M NaBH_4_ solutions. Reduction in 1 M NaBH_4_ (LPR 1 M) gave a markedly lower activity (44.6%, 11.6%), even below that of the commercial catalyst, whereas reduction in 5 M NaBH_4_ (LPR 5 M, 57.3% and 23.4%) increased the activity markedly relative to LPR 1 M, reaching a level comparable to that of the standard LPR catalyst prepared in 3 M solution. Hence, liquid-phase reduction becomes effective only above a threshold reductant concentration, beyond which the activity saturates. Notably, the gap between the NH_3_ conversion and the H_2_ yield remains close to 33% for all eight catalysts in Fig. S6, confirming that the supplied O_2_ is fully consumed through complete combustion of NH_3_ regardless of the catalyst, in agreement with the FT-IR results in Fig. S4.

### 3.4 Adsorption–desorption properties of catalysts

To examine whether the state of the support affects the desorption of nitrogen from Ru, N2-TPD was carried out (Fig. 4a). All three catalysts show a desorption peak above 350 °C, assigned to recombinative desorption of nitrogen from Ru.^43^ The peak temperatures are 430 °C (LT), 470 °C (HT) and 370 °C (LPR), so that N_2_ desorbs most readily from LPR. This ordering (LPR < LT < HT) coincides with the ordering of the surface Ce^3+^/Ce^4+^ ratio (Table 1) and with the ordering of the ammonia decomposition activity (Fig. 1b), indicating that the strength with which nitrogen is bound to the Ru surface differs among the catalysts and correlates with their activity. Since recombinative N_2_ desorption is widely accepted as the rate-limiting step of ammonia decomposition, we interpret this correlation as indicating that Ru in contact with the vacancy-rich support binds nitrogen more weakly, as expected for electron donation from the support.^47,48^ This interpretation rests on the observed correlation rather than on a direct kinetic determination; measurements of reaction orders and apparent activation energies under differential conditions would be required to establish it directly.

**Fig. 4:**
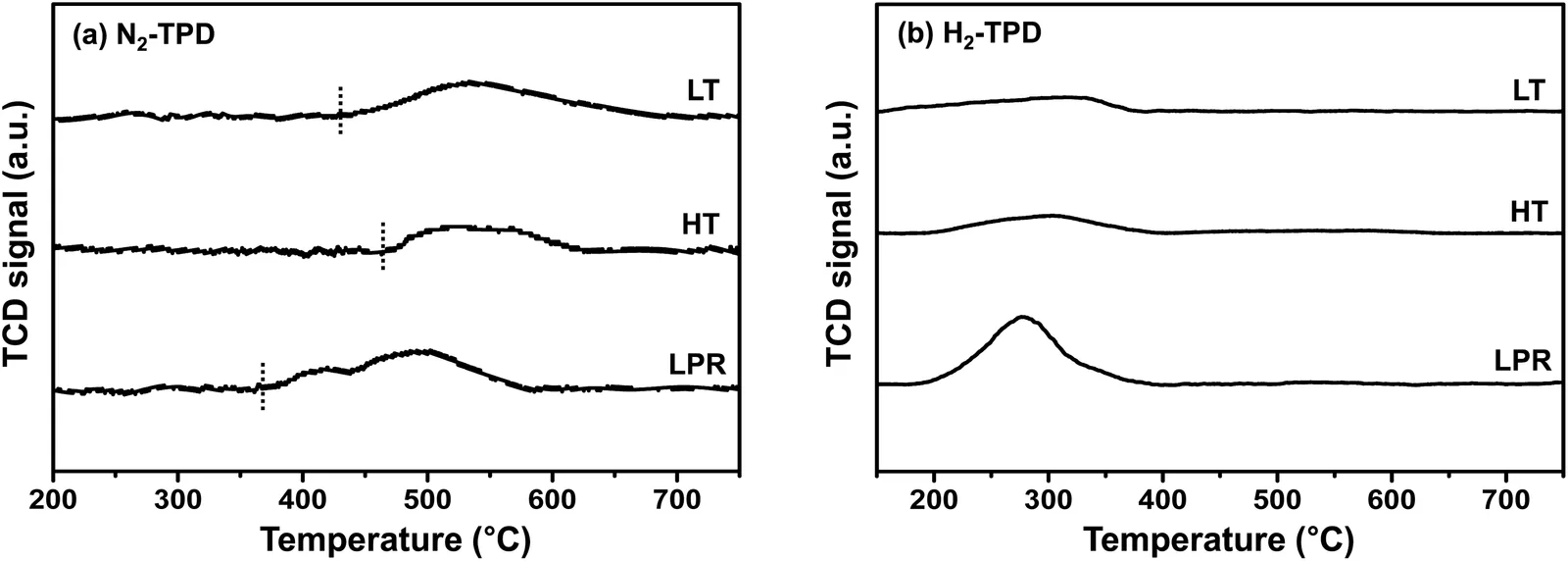
(a) N_2_-TPD and (b) H_2_-TPD profiles of the Ru/CeO_2_ catalysts.

Furthermore, to confirm the enhanced activity facilitated by oxygen vacancies through an additional mechanism, namely hydrogen spillover, we conducted analyses using H_2_-TPD and pulse chemisorption techniques with H_2_ and CO (Fig. 4b and Table 2). Fig. 4b shows that the LPR catalyst exhibits a broader and larger peak area compared to the other catalysts, indicating a higher amount of hydrogen desorption. The Ru particle sizes of the LT and LPR catalysts are statistically identical (12.4 ± 0.7 and 12.9 ± 0.9 nm), although their CO uptakes differ by a factor of about 1.5 (Table 2). The substantially higher H_2_ desorption of LPR is consistent with significant hydrogen spillover; because part of this difference could in principle reflect the difference in exposed Ru sites, spillover is assessed below through the comparative indicator *Q*_spillover_ = 2*Q*_H_2__ − *Q*_CO_, in which the contribution of the CO-titrated Ru sites is subtracted. The peak detected in the H_2_-TPD analysis is ascribed to the desorption of H_2_ adsorbed on the Ru surface and at neighboring oxygen vacancies.^44^ Typically, H_2_ preferentially adsorbs on Ru and does not directly adsorb on CeO_2_. However, when oxygen vacancies are generated, H_2_ dissociatively adsorbed on Ru can spill over to neighboring oxygen vacancies.^29^ The observed peak in LPR indicates hydrogen spillover, likely due to the high density of oxygen vacancies in the vicinity of Ru nanoparticles.

**Table 2:** Results of CO and H_2_ pulse chemisorption and quantity of hydrogen spillover

Catalyst	*Q*_CO_ a (10^−2^ cm^3^ g^−1^ STP)	*Q*_H_2__ b (10^−2^ cm^3^ g^−1^ STP)	*Q*_Spillover_ c (10^−2^ cm^3^ g^−1^ STP)
LT	1.4	3.65	5.9
HT	0.5	0.25	∼0
LPR	2.1	4.9	7.7

a Quantity of CO uptake in CO pulse chemisorption analysis.

b Quantity of H_2_ uptake in H_2_ pulse chemisorption analysis.

c Quantity of hydrogen spillover, *Q*_spillover_ = 2*Q*_H_2__ − *Q*_CO._

The results from the H_2_ and CO-pulse chemisorption in Table 2 illustrate the degree of hydrogen spillover, represented by comparing the adsorption quantity of H_2_ and CO. Typically, CO is known to adsorb in a 1 : 1 ratio with Ru atoms, while H_2_ undergoes dissociation on ruthenium, resulting in the adsorption of one H_2_ molecule onto two Ru atoms. In cases where hydrogen spillover occurs during H_2_ chemisorption, it could potentially indicate a larger ruthenium surface area compared to CO chemisorption analysis, where spillover does not take place.^50^ In the case of the HT catalyst, the molar quantity of ruthenium bound to CO was similar to that bound to H, whereas with the LPR and LT catalysts, a significantly higher molar quantity of Ru bound to H was evident. This trend is more pronounced in the LPR catalyst. Consequently, this difference is reflected in the quantity of hydrogen spillover (*Q*_spillover_). Therefore, the order of magnitude of *Q*_spillover_ aligns with the order of oxygen vacancy abundance: LPR > LT > HT. This correlation implies that the catalyst's oxygen vacancies could trigger hydrogen spillover effect.

Under steady-state reaction conditions, this does not correspond to a continuous accumulation of hydrogen on CeO_2_. Hydrogen transferred to the vacancy sites is released by recombinative desorption from the support, a behavior reported for oxide promoters that accept and readily release spilled-over hydrogen,^51^ so that the vacancy sites are continuously regenerated. The catalytic consequence is therefore a redistribution of hydrogen between the metal and the support: the availability of additional sites capable of accommodating hydrogen lowers the steady-state coverage of hydrogen on Ru, leaving more sites available for NH_3_ adsorption and dehydrogenation and thereby mitigating hydrogen poisoning of the active sites. Likewise, the correlation between *Q*_spillover_, the oxygen vacancy concentration and the catalytic activity is presented here as evidence of a consistent trend among the three catalysts. The proposed effect of spillover on the steady-state hydrogen coverage of Ru is an interpretation of this trend and has not been verified by direct kinetic measurement in the present work.

### 3.5 Thermodynamic and techno-economic analysis

To assess the thermodynamic feasibility and economic potential of the autothermal oxidative decomposition of ammonia (ODA), a comprehensive evaluation was conducted integrating standard-state thermochemical analysis with a concept-level process model.

#### 3.5.1 Thermodynamic evaluation

Based on these data, the changes in standard enthalpy (Δ*H*°), entropy (Δ*S*°), and Gibbs free energy (Δ*G*°) were calculated for the two governing reactions: ammonia decomposition (2NH_3_ → N_2_ + 3H_2_) and total oxidation (4NH_3_ + 3O_2_ → 2N_2_ + 6H_2_O). Detailed reaction stoichiometries, normalization methods, and the derivation of the autothermal heat balance equation are provided in SI Material: Section 2.

As summarized in Table 3, the decomposition of ammonia is endothermic (Δ*H*° = +92.22 kJ mol^−1^) and non-spontaneous at standard conditions 
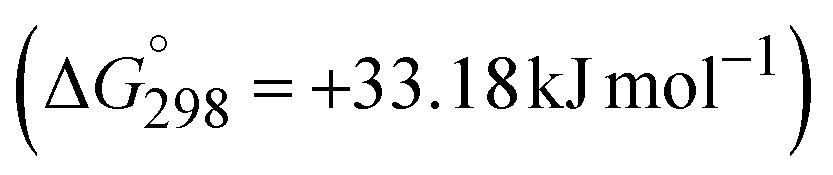
. Conversely, the oxidation of ammonia is highly exothermic (Δ*H*° = −1266.48 kJ mol^−1^) and thermodynamically spontaneous 
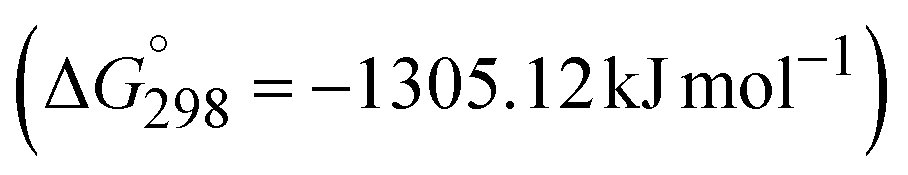
. All values are given per mole of reaction as written. Fig. 5 illustrates the temperature dependence of Gibbs free energy. The profile indicates that while oxidation remains favorable across the entire temperature range (298–1200 K), ammonia decomposition only becomes thermodynamically favorable at temperatures exceeding approximately 460 K.

**Table 3:** Standard-state reaction properties at 298 K, given per mole of reaction as written

No.	Reaction	Δ*H*° (kJ mol^−1^)	Δ*S*° (J mol^−1^ K^−1^)	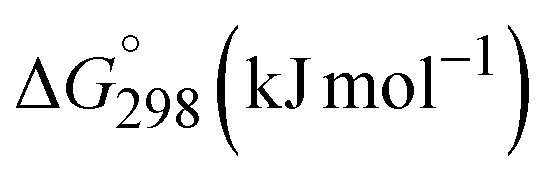
(1)	2NH_3_ → N_2_ + 3H_2_	+92.22	+198	+33.18
(2)	4NH_3_ + 3O_2_ → 2N_2_ + 6H_2_O	−1266.48	+130	−1305.12

**Fig. 5:**
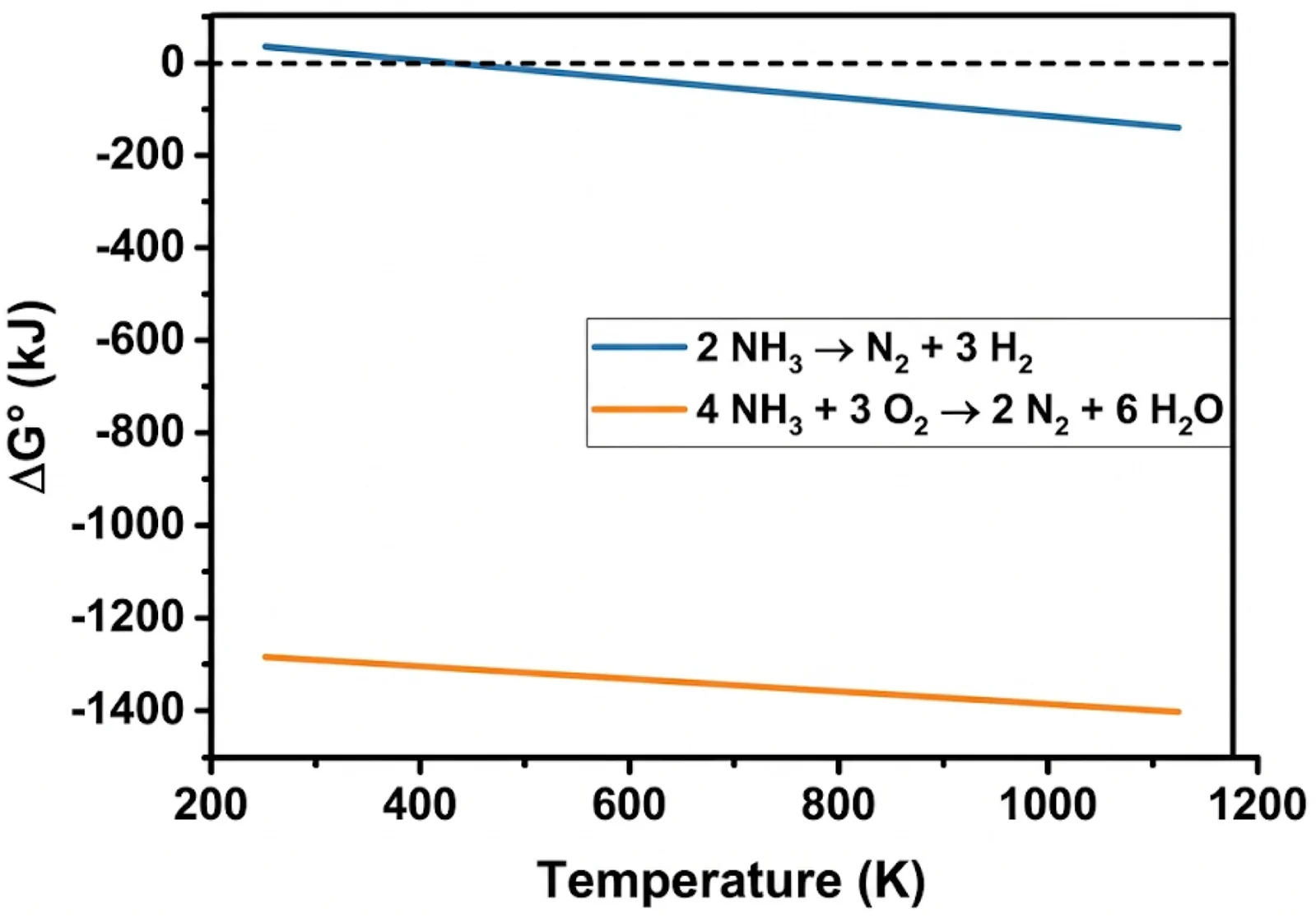
Standard Gibbs free energy change for ammonia decomposition and oxidation of ammonia as a function of temperature.

Autothermal operation relies on balancing the endothermic heat demand of decomposition with the exothermic heat released from partial oxidation. An adiabatic heat-balance calculation determined the critical oxidation fraction (*α*) to be 0.127, which corresponds to a stoichiometric O_2_/NH_3_ ratio of 0.095 (Table 4). Fig. S7 shows the calculated heat surplus as a function of the O_2_/NH_3_ feed ratio, illustrating the transition from endothermic to autothermal and exothermic operation. In this study, the experimental feed ratio was fixed at NH_3_/O_2_ = 4 (O_2_/NH_3_ = 0.25). Under these conditions, the oxidation fraction is approximately 2.6 times higher than the theoretical minimum required for autothermal balance. This excess oxidation generates a net heat release of approximately 75 kJ mol^−1^-NH_3_. This surplus heat effectively drives the self-sustaining ignition observed near 455 K and maintains the stable temperature profile over the liquid-phase-reduced Ru/CeO_2_ (LPR) catalyst (Fig. S2). It is noteworthy that the theoretical maximum hydrogen yield under these autothermal conditions is limited to 66.7%. The LPR catalyst achieved a yield of 26.4%, which corresponds to roughly 40% of the thermodynamic limit. This gap suggests that there is substantial room for further enhancing hydrogen productivity through precise oxygen control and the engineering of oxygen vacancies to promote decomposition kinetics.

**Table 4:** Adiabatic heat-balance results

Parameter	Value
Heat of decomposition per NH_3_	+46.11 kJ mol^−1^
Heat of oxidation per NH_3_	−316.62 kJ mol^−1^
Required NH_3_ oxidation fraction, *α*	0.127
Corresponding O_2_/NH_3_ ratio	0.095

#### 3.5.2 Preliminary techno-economic assessment

A concept-level techno-economic analysis (TEA) was performed to estimate the Levelized Cost of Hydrogen (LCOH) for the autothermal ODA process. Table 5 details the key economic assumptions used in the model. The baseline scenario assumes an ammonia feedstock price of $400 per ton (2024–2025 average) and the experimental single-pass H_2_ yield of the LPR catalyst (26.4%), for which the calculated LCOH is $9.6 kg^−1^-H_2_; raising the yield to 33% (50% of the ceiling) lowers the LCOH to $7.74 kg^−1^-H_2_. A breakdown of the cost components reveals that the ammonia feedstock is the dominant cost driver, accounting for 89% ($8.53 kg^−1^-H_2_) of the total LCOH. The remaining costs are distributed among oxygen supply ($0.50 kg^−1^-H_2_), catalyst replacement ($0.21 kg^−1^-H_2_), electricity consumption ($0.20 kg^−1^-H_2_), and capital recovery ($0.16 kg^−1^-H_2_).

**Table 5:** Key economic assumptions for a concept-level techno-economic analysis

Item	Value	Basis
Ammonia price	$400 per ton	2024–25 average^52^
Oxygen price	$50 per ton	Small PSA unit^53^
Single-pass H_2_ yield	26.4% (39.6% of 66.7%)	Experimental (this work); 33% and 66.7% as improvement scenarios
Net process heat duty	0	Autothermal
Ru price	$15 per g	Long-term average^54^
Catalyst	5 wt% Ru/CeO_2_, 3 years life, 3.3 kg-H_2_ kg·cat^−1^ day^−1^	Design value at the 26.4% base-case yield
Electricity	2.5 kWh kg H_2_^−1^@$0.08 kWh^−1^	Compression + O_2_ PSA^55,56^
CAPEX	$500 kg^−1^-H_2_ day installed; 15 years, 8% WACC	Concept estimate^57,58^

Fig. 6 presents the results of a two-factor sensitivity analysis, examining the impact of ammonia price ($300–500 per ton) and single-pass H_2_ yield (26.4–67%) on the LCOH. While the base case yields a cost of $9.6 kg^−1^-H_2_, the analysis shows that the LCOH can vary significantly, ranging from approximately $3.2 kg^−1^-H_2_ (at $300 per ton and the 66.7% ceiling) to $12.4 kg^−1^-H_2_ (at $500 per ton and the demonstrated 26.4% yield). Raising the single-pass yield to 33%, half of the theoretical limit, already lowers the LCOH to $7.74 kg^−1^-H_2_ at the base ammonia price, and increasing the yield further towards the theoretical limit (66.7%) drastically reduces the production cost. This analysis highlights that catalyst performance improvement through vacancy engineering is, alongside ammonia cost reduction, the primary lever for reducing the cost of hydrogen production from ammonia.

**Fig. 6:**
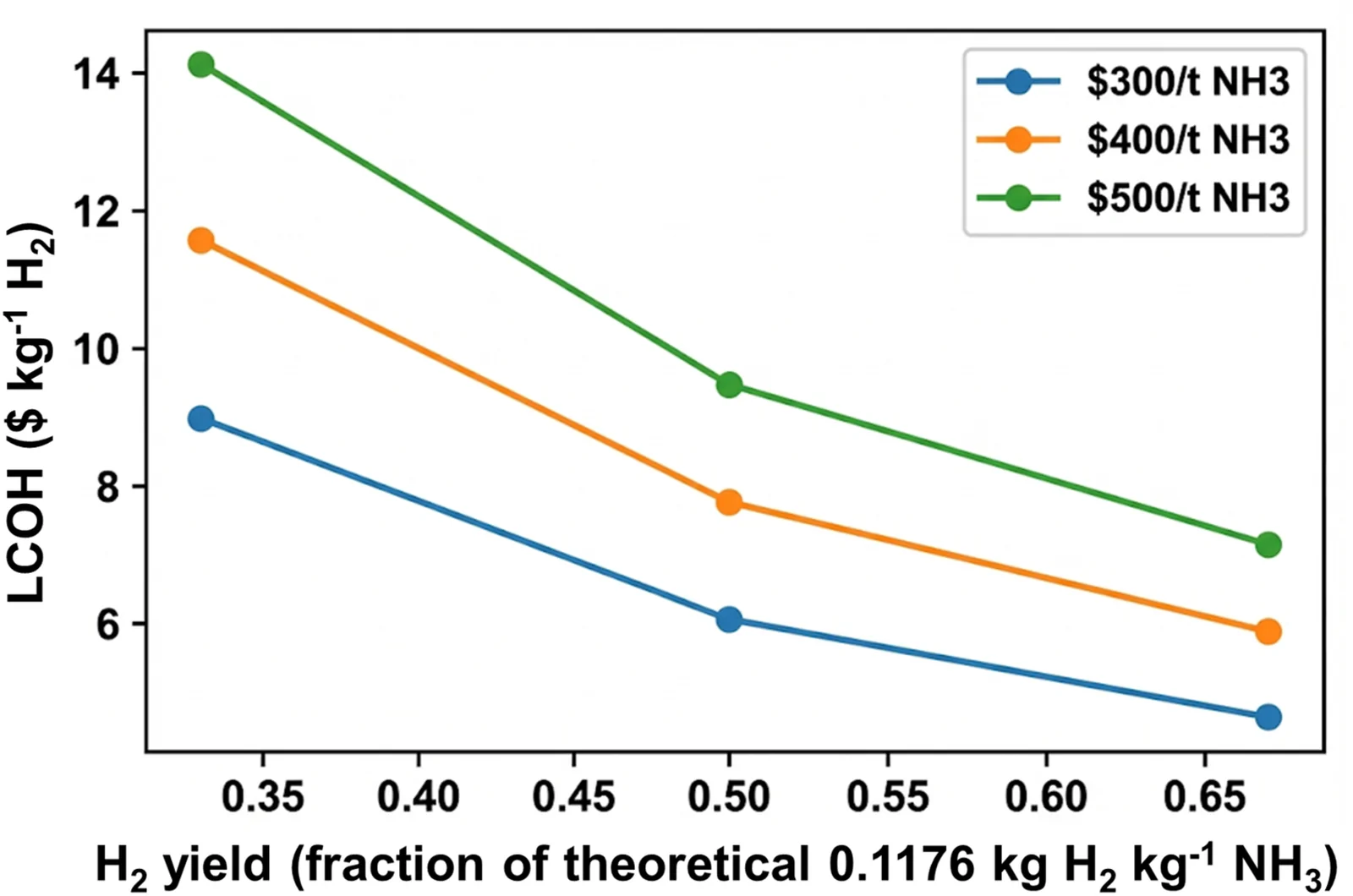
LCOH as a function of ammonia price ($300–500 per ton) and single-pass hydrogen yield (26.4–67% of the 0.1776 kg-H_2_ kg^−1^ NH_3_ theoretical value); the lower bound of the yield axis corresponds to the single-pass yield experimentally demonstrated in this work.

## 4 Conclusion

The Ru/CeO_2_ catalyst for oxidative decomposition of ammonia was synthesized by wet impregnation method, and reduced by thermal reduction and liquid-phase reduction method. The reduced catalysts demonstrated maximum attainable activity in oxidation reaction of ammonia, and the H_2_ yield in ODA was predominantly determined by the ammonia decomposition activity of the catalyst. The catalyst, pretreated by liquid-phase reduction, effectively generated oxygen vacancies, as shown by the Ce^3+^/Ce^4+^ ratio (XPS) and the Ce M_4,5_ white-line ratio (EELS). N_2_-TPD showed that nitrogen desorbs from the vacancy-rich LPR catalyst at the lowest temperature, which we interpret as weakened Ru–N binding at the rate-determining recombinative desorption step, and H_2_-TPD together with CO/H_2_ chemisorption indicated enhanced hydrogen spillover to the support. Moreover, the enhanced hydrogen spillover might prevent hydrogen poisoning on the ruthenium surface, facilitating efficient ammonia decomposition. According to XRD and TEM analysis, unlike thermal reduction, liquid-phase reduction did not induce sintering of the active metal, thereby preserving metal dispersion.

On the other hand, thermodynamic analysis confirmed that the ODA reaction can operate autothermally when approximately 12–13% of the fed ammonia is oxidized, balancing the endothermic decomposition and exothermic oxidation reactions. The experimental oxygen ratio (NH_3_/O_2_ = 4) ensures a modest excess of heat, explaining the self-sustaining behavior of the system. Furthermore, a preliminary techno-economic analysis estimated an LCOH of ∼$9.6 kg^−1^-H_2_ at the experimental yield, decreasing to ∼$7.7 kg^−1^-H_2_ at a 33% yield, primarily determined by ammonia feedstock cost, and projected significant cost reductions through catalyst performance optimization or coupling with renewable ammonia production.

Therefore, the liquid-phase reduction with NaBH_4_ effectively induced oxygen vacancies, enhancing the activity of the oxidative decomposition of ammonia from 20.6% of LT catalyst to 26.4% of LPR catalyst in H_2_ yield. The NaBH_4_ liquid-phase reduction strategy may also benefit other oxide-supported metal catalysts where vacancy engineering is desirable.

Direct verification of the proposed mechanism, through measurements of reaction orders and apparent activation energies under differential conditions, remains a subject for future work.

## Author contributions

Soohang Lee: conceptualization, methodology, investigation, data curation, visualization, writing – original draft. Seok-Ho Lee: conceptualization, methodology, investigation, data curation, visualization, writing – original draft. Minseon Park: methodology, investigation, data curation. Tae Hyun Kim: data curation, visualization. In Hye Kwak: methodology, investigation, data curation, visualization. Hyeon Ih Ryu: methodology, investigation, data curation, visualization. Na Young Kim: investigation, methodology, writing – review & editing. Jin Suk Chung: writing – review & editing, funding acquisition. Byung Jin Lee: investigation, validation, supervision, writing – review & editing. Kwan-Young Lee: project administration, validation, supervision, funding acquisition.

## Conflicts of interest

There are no conflicts to declare.

## Abbreviations

XRD: X-ray diffraction
TEM: Transmission electron microscopy
XPS: X-ray photoelectron spectroscopy
H_2_-TPR: Temperature-programmed reduction with hydrogen
N_2_-TPD: Temperature-programmed desorption of nitrogen
H_2_-TPD: Temperature-programmed desorption of hydrogen
TCD: Thermal conductivity detector
DI water: Deionized water

## Supplementary Material

RA-OLF-D6RA06193A-s001

## Data Availability

The data supporting this article have been included as part of the supplementary information (SI). Additional data are available from the corresponding authors upon reasonable request. Supplementary information is available. See DOI: https://doi.org/10.1039/d6ra06193a.
