# Supplementary material for: Autothermal hydrogen production via oxidative decomposition of ammonia over vacancy-engineered Ru/CeO2 prepared by NaBH4 liquid-phase reduction
Source: RSC Adv. 2026 Oct 5;16(46):60026–40. doi: 10.1039/d6ra06193a (PMC13635995; doi:10.1039/d6ra06193a)
Supplement: Supplementary file 1 [file RA-016-D6RA06193A-s001.pdf]

Supplementary Information (SI) for RSC Advances.
This journal is © The Royal Society of Chemistry

Supplementary Materials

Autothermal Hydrogen Production via Oxidative Decomposition of Ammonia over Vacancy-Engineered Ru/CeO_2 Prepared by $NaBH_4$ Liquid-Phase Reduction

Soohang Lee,[1,†] Seok-Ho Lee,[1,2,†] Minseon Park,[3,†] Tae Hyun Kim,[1] In Hye Kwak,[4] Hyeon Ih Ryu,[4] Na Young Kim,[1] Jin Suk Chung,[3] Byung Jin Lee,[3,*] and Kwan-Young Lee [1,2,*]

[1] Department of Chemical and Biological Engineering, Korea University, 145 Anam-ro, Seongbuk-gu, Seoul 02841, Republic of Korea

[2] Strategic Research Center for Clean Hydrogen Storage and Utilization Korea Institute of Science and Technology, Seoul 02792, Republic of Korea

[3] School of Energy and Chemical Engineering, University of Ulsan, 93 Daehak-ro, Nam-gu, Ulsan 44610, Republic of Korea

[4] Strategic Research Center for Smart Battery, Korea Basic Science Institute, Daejeon 34133, Korea

† These authors contributed equally to this work.

* Corresponding author:

Byung Jin Lee – Email: byungjlee@ulsan.ac.kr

Kwan-Young Lee – Email: kylee@korea.ac.kr

Section I: Supplementary figures and tables

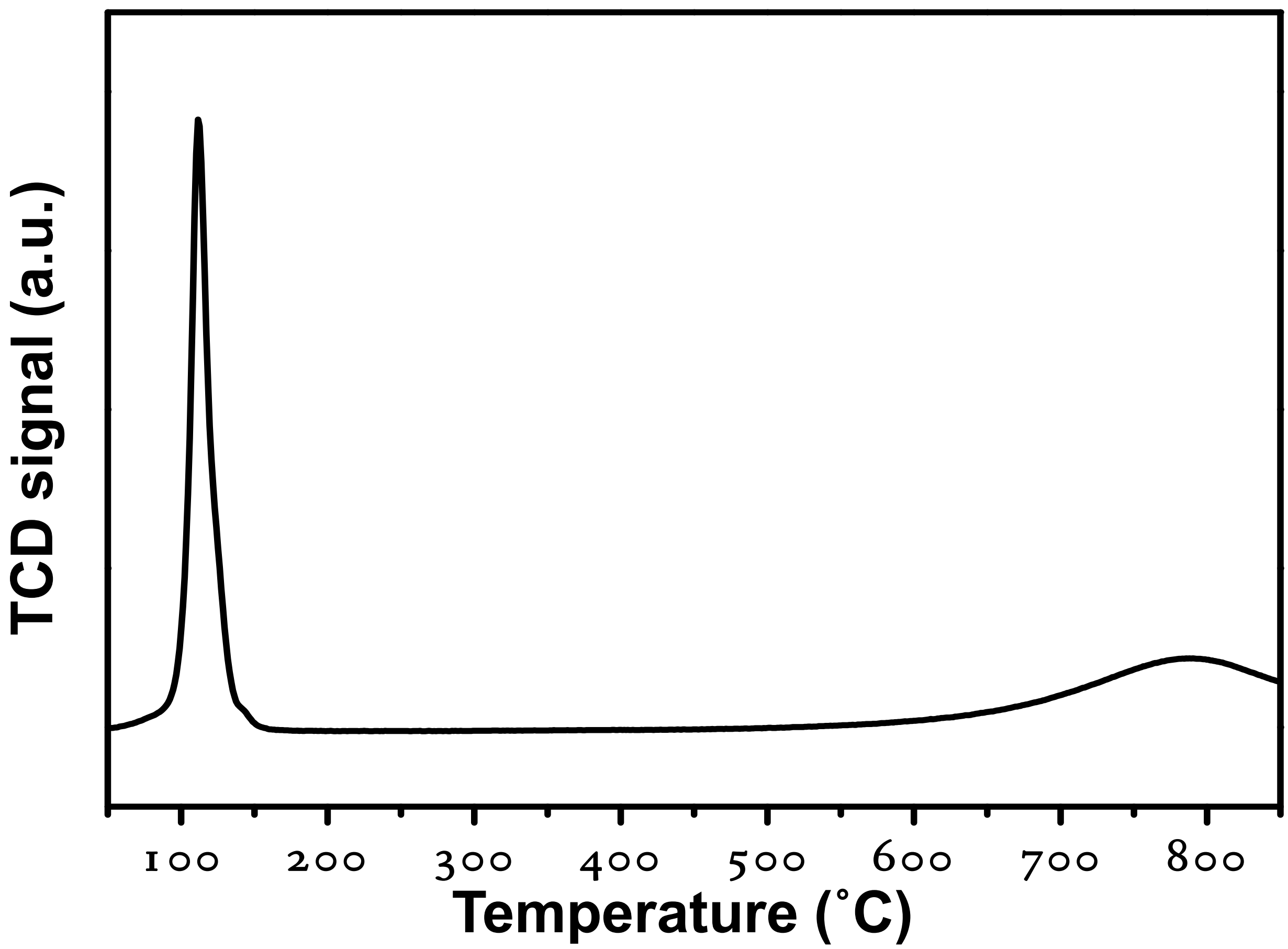

Figure S1. H_2-TPR profile of Ru/CeO_2 catalyst.

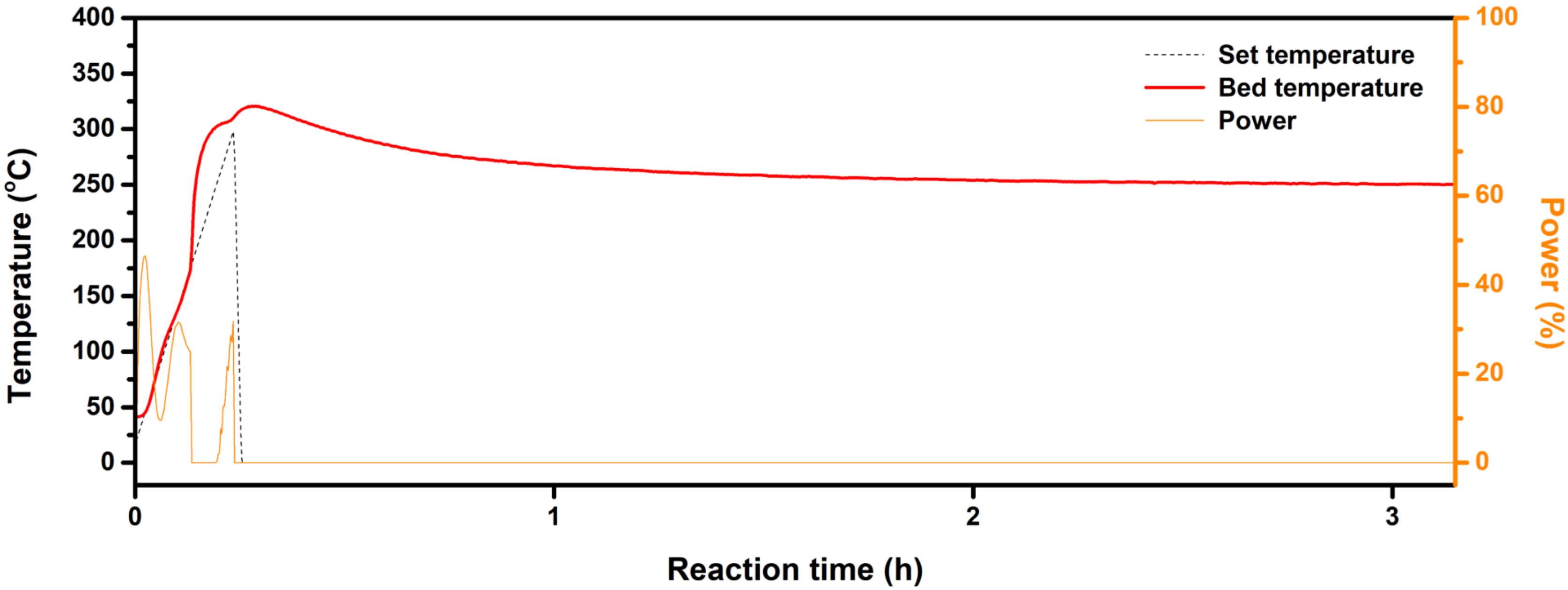

Figure S2. Time-dependent temperature profile of the catalyst bed during oxidative decomposition of ammonia over the liquid-phase-reduced Ru/CeO_2 (LPR) catalyst. External heating was limited to the 20 °C·min^{-1} ramp to the 300 °C set point, completed within ca. 15 min of start-up; the furnace power trace is zero thereafter.

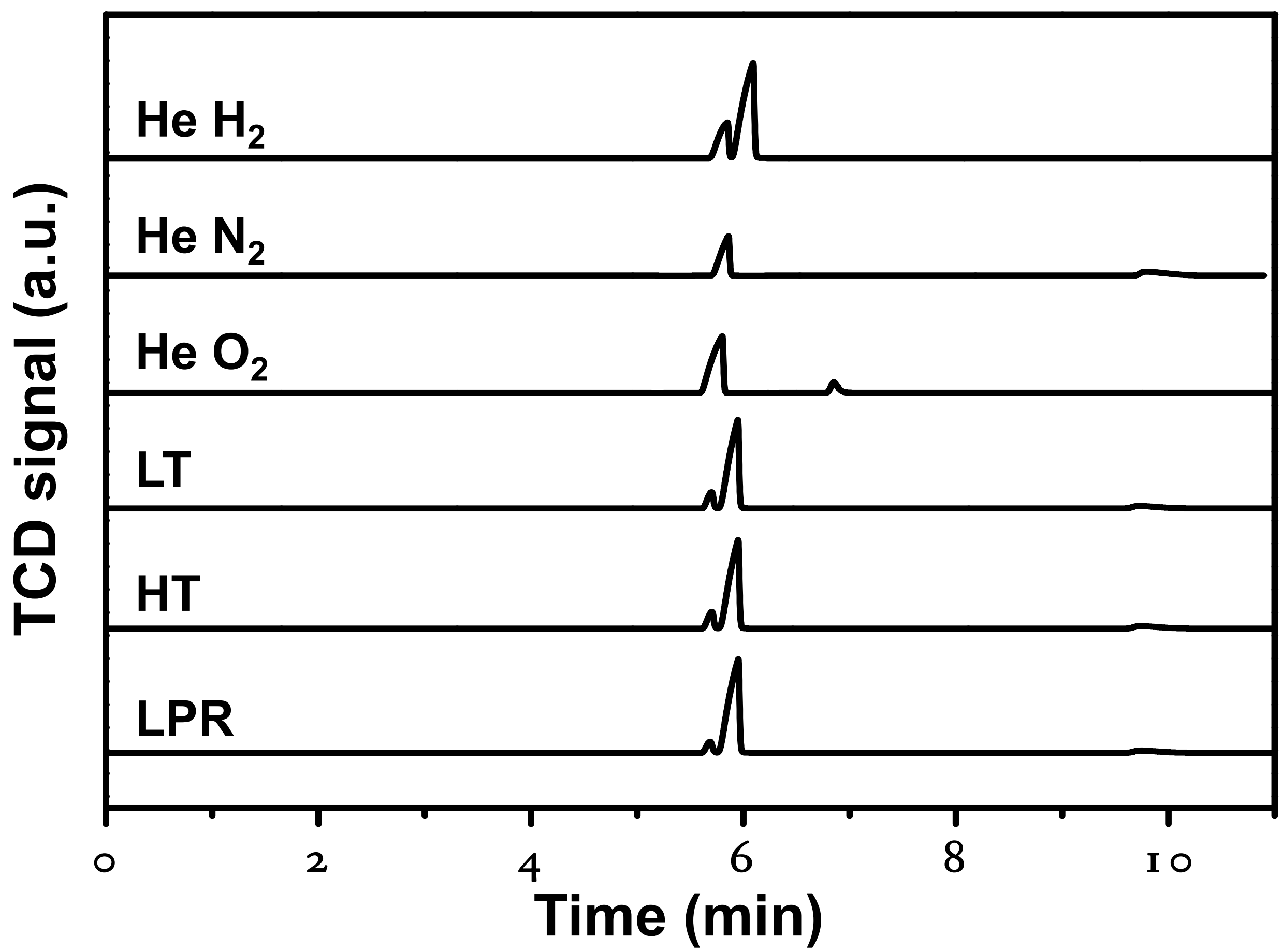

Figure S3. Gas chromatogram for various feed gases composition (He-H_2, He-N_2, and He-O_2) and effluent gases using each Ru/CeO_2 catalyst in oxidative decomposition reaction of ammonia (LT, HT, and LPR).

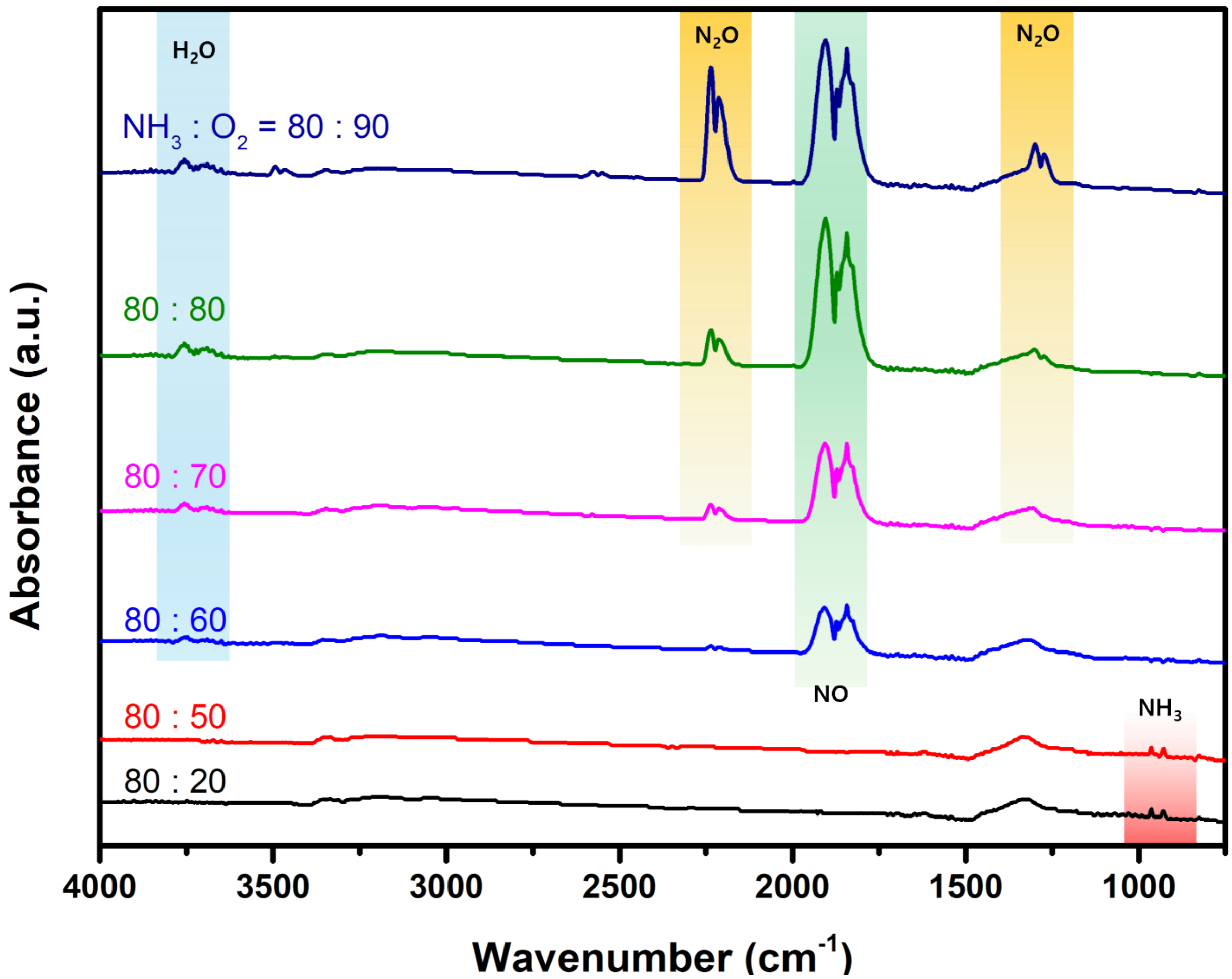

Figure S4. FT-IR spectra of the reactor effluent obtained at different NH_3:O_2 feed ratios (80:20, 80:50, 80:60, 80:70, 80:80 and 80:90 ml·min^{-1}) over a commercial 5 wt% Ru/Al_2O_3 catalyst. Absorption bands assigned to NH_3, H_2O, NO and N_2O are indicated. The feed composition used throughout this study corresponds to NH_3:O_2 = 80:20.

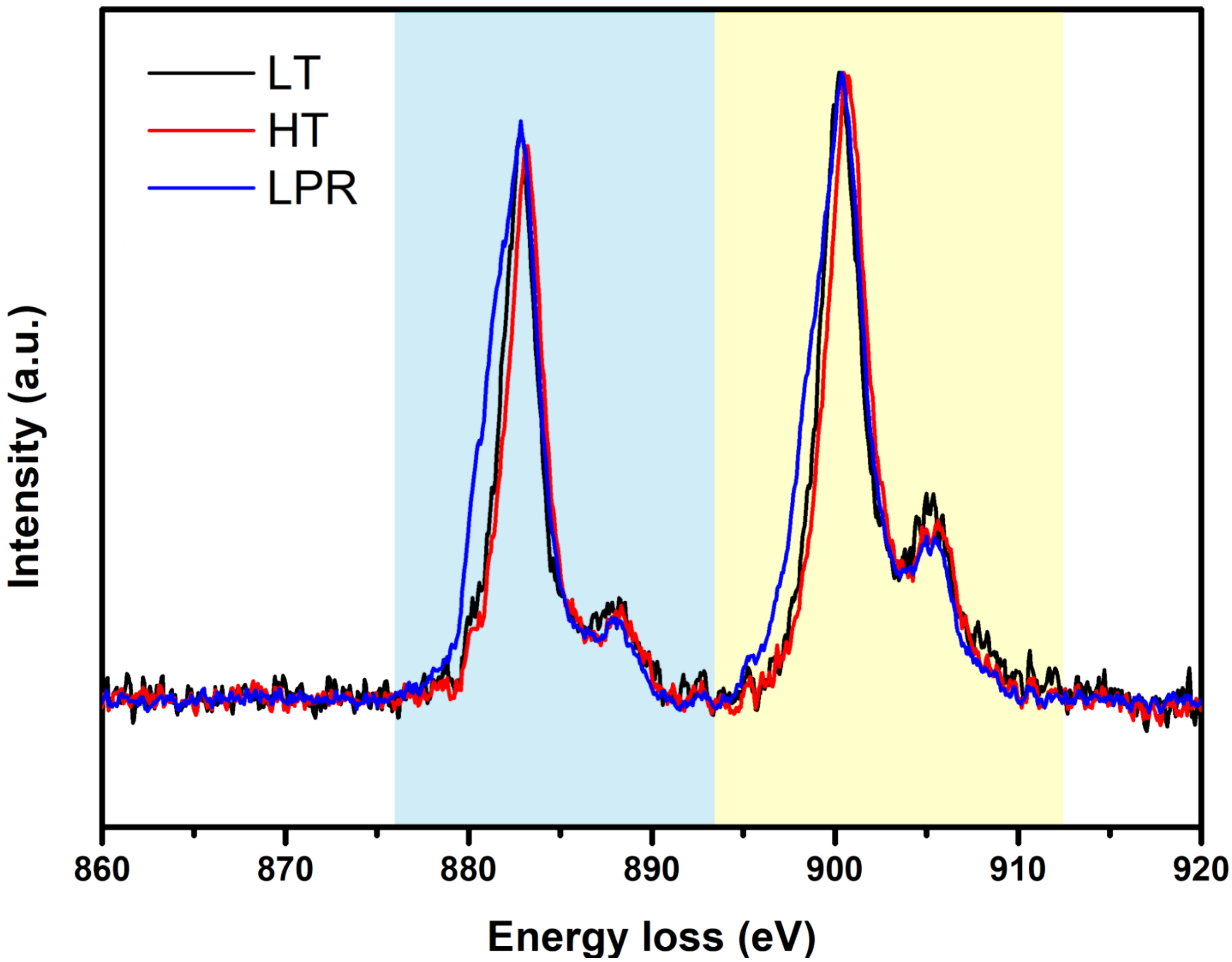

Figure S5. Ce $M_{4,5}$ edge EELS spectra of the LT, HT and LPR catalysts, after background subtraction and normalization. The M_5 (blue section) and M_4 (yellow section) white lines appear at approximately 883 and 900 eV, respectively.

Table S1. Integrated white-line intensity ratio of the Ce $M_{4,5}$ edge for the Ru/CeO_2 catalysts.

Catalysts	IM_5/IM_4 [a]
LT	0.708
HT	0.710
LPR	0.786

[a] Determined from the spectra in **Figure S5** by integrating the M_5 white line over 876–893 eV and the M_4 white line over 893–912 eV, using the same limits for all three catalysts. A higher ratio indicates a greater abundance of Ce^{3+}.

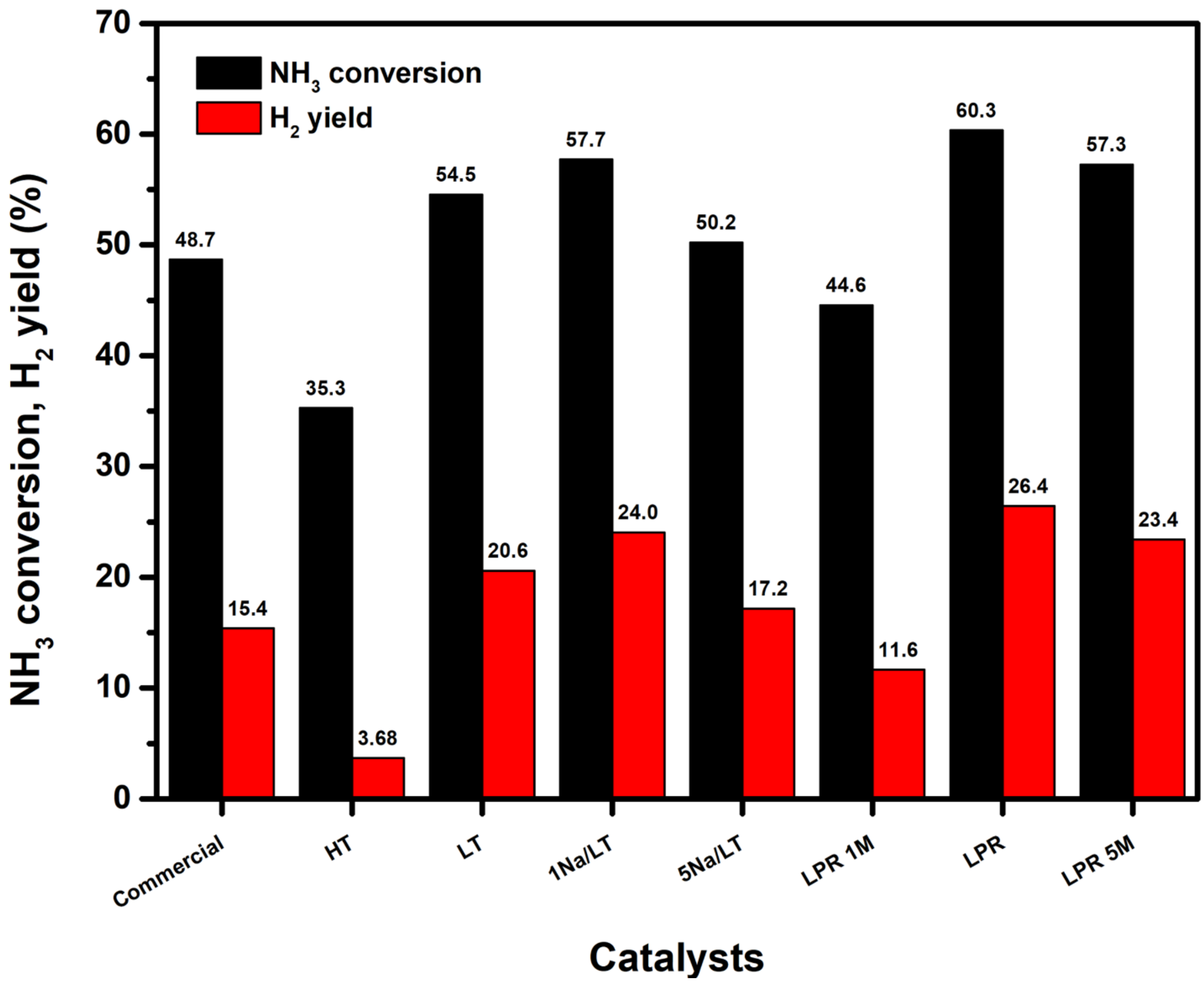

Figure S6. NH_3 conversion and H_2 yield in the oxidative decomposition of ammonia over the control catalysts listed in **Table S2**. Reaction conditions are identical to those of **Figure 1a**; the HT, LT and LPR data are those of **Figure 1a**.

Table S2. Residual Na and B contents and catalytic performance of the control catalysts in the oxidative decomposition of ammonia.

Catalysts	Preparation	Na (wt.%) [a]	B (wt.%) [a]	NH_3 conversion (%) [b]	H_2 yield (%) [b]
Commercial	5 wt% Ru/Al_2O_3	-	-	48.7	15.4
HT	H_2 reduction, 900 °C	-	-	35.3	3.68
LT	H_2 reduction, 250 °C	-	-	54.5	20.6
1Na / LT	LT+1wt% Na impregnation	0.62	-	57.7	24.0
5Na / LT	LT+5wt% Na impregnation	3.34	-	50.2	17.2
LPR 1M	1M $NaBH_4$	0.02	0.09	44.6	11.6
LPR	3M $NaBH_4$	0.02	0.07	60.3	26.4
LPR 5M	5M $NaBH_4$	0.02	0.16	57.3	23.4

[a] Determined by ICP-OES after acid digestion.

[b] NH_3:O_2:He = 80:20:5 ml·min^{-1}, 50 mg of catalyst (GHSV = 126,000 ml·h^{-1}g$_{cat}^{-1}$), autothermal operation after ignition, 3 h on stream (**Figure S6**). HT, LT and LPR data are those of **Figure 1a**.

Section II: Supplementary information of thermodynamical evaluation

1. Reaction basis and normalization of thermodynamic quantities

All thermodynamic and heat-balance calculations in this study are performed on a clearly defined reaction basis to avoid ambiguity in energy normalization. **Table S3** summarizes the reaction stoichiometries used for ammonia decomposition and ammonia oxidation, together with the corresponding normalization per mole of NH_3.

Table S3. Reaction basis and normalization used in this study

Reaction	Stoichiometric equation	$\Delta H^\circ_{2\square\square}$ (reaction basis)	Normalization
NH_3 decomposition	$2\ NH_3 \rightarrow N_2 + 3\ H_2$	+92.22 kJ mol^{-1}	+46.11 kJ mol^{-1} NH_3
NH_3 oxidation	$4\ NH_3 + 3\ O_2 \rightarrow 2\ N_2 + 6\ H_2O(g)$	−1266.5 kJ mol^{-1}	−316.62 kJ mol^{-1} NH_3

Standard reaction enthalpies were calculated from standard heats of formation at 298 K using

$\Delta H^\circ = \Sigma \nu_i \Delta H^\circ_{f,i}$ where ν_i denotes stoichiometric coefficients. The values normalized per mole of NH_3 are used consistently throughout the autothermal heat-balance and techno-economic analyses.

2. General formulation of autothermal heat balance

For autothermal operation, the heat released by partial oxidation of ammonia must exactly offset the endothermic heat required for ammonia decomposition. Let □ denote the fraction of NH_3 undergoing oxidation,

while (1−□) undergoes decomposition. Under adiabatic conditions, the heat balance per mole of NH_3 feed is expressed as

$$□\left(□H_{ox}^{NH_3}\right) + (1 - □)\left(□H_{dec}^{NH_3}\right) = 0$$

where

$$□H_{dec}^{NH_3} = +46.11 \text{ kJ/mol}$$

$$□H_{ox}^{NH_3} = -316.62 \text{ kJ/mol}$$

Solving for □ yields

$$□ = \frac{□H_{dec}^{NH_3}}{-□H_{ox}^{NH_3} + □H_{dec}^{NH_3}} = 0.127$$

Thus, oxidation of 12.7% of the ammonia feed is sufficient to render the process thermally neutral under ideal adiabatic conditions. Using the stoichiometric ratio of $4NH_3{:}3O_2$ for oxidation, the corresponding minimum oxygen requirement is

$$\left(\frac{O_2}{NH_3}\right)_{auto} = 0.75□ = 0.095$$

This value represents the theoretical lower bound for autothermal operation.

3. Oxygen ratio versus heat surplus

In the experimental configuration, an NH_3/O_2 feed ratio of 4 (equivalent to $O_2/NH_3 = 0.25$) was employed, which exceeds the autothermal requirement by a factor of approximately 2.6. The resulting heat surplus per mole of NH_3 is given by

$$Q_{surplus} = -[\square_{exp} \square H_{ox}^{NH_3} + \left(1 - \square_{exp}\right) \square H_{dec}^{NH_3}]$$

where

$$\square_{exp} = \frac{(O_2/NH_3)_{exp}}{0.75}$$

At $O_2/NH_3 = 0.25$, the heat balance gives Qsurplus = −75 kJ mol^{-1} NH_3. Under the sign convention of the equation above, a negative value denotes net heat release, so this corresponds to a net release of approximately 75 kJ mol^{-1} NH_3. This net exothermicity explains the experimentally observed spontaneous ignition near 455 K and the sustained temperature profile in the catalyst bed (**Figure S2**), providing thermodynamic justification for the self-sustaining behavior of the ODA process.

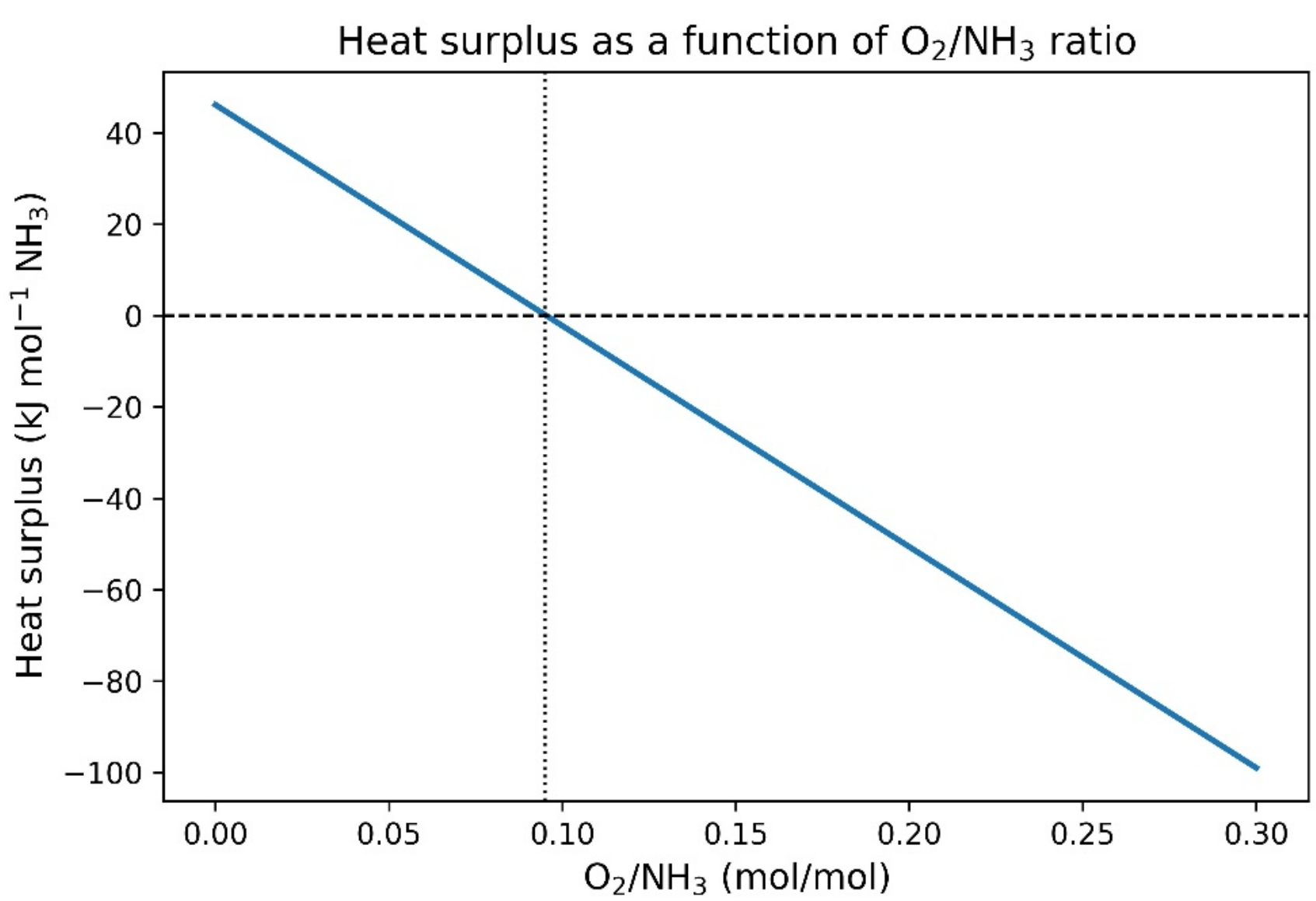

Figure S7. Calculated heat surplus as a function of O_2/NH_3 feed ratio, showing the transition from endothermic to autothermal and exothermic operation. The dashed line indicates the autothermal condition ($O_2/NH_3 = 0.095$).
